# GC–MS metabolomic profiling and PPARγ-targeted *in silico* approaches for identifying a potential anti-diabetic compound from traditional rice varieties

**DOI:** 10.3389/fnut.2026.1800615

**Published:** 2026-05-05

**Authors:** Saranya Nallusamy, K. Vanitha, M. Riswana Begam, N. K. Sathyamoorthy, P. Geetha, S. Parveen, J. Akhansha, G. Senbagavalli, S. Kokilavani

**Affiliations:** 1 Department of Plant Molecular Biology and Bioinformatics, Tamil Nadu Agricultural University, Coimbatore, India; 2 Department of Fruits Science, Tamil Nadu Agricultural University, Coimbatore, India; 3 Agro Climate Research Centre, Directorate of Crop Management, Tamil Nadu Agricultural University, Coimbatore, India; 4 Centre for Post-Harvest Technology, Agricultural Engineering College and Research Institute, Tamil Nadu Agricultural University, Coimbatore, India; 5 Department of Food Process Engineering, Agricultural Engineering College and Research Institute, Tamil Nadu Agricultural University, Coimbatore, India

**Keywords:** anti-diabetic activity, GC–MS, molecular docking, molecular dynamics simulation, PPARγ, traditional rice varieties

## Abstract

Traditional rice varieties are known to exhibit anti-oxidant, anti-inflammatory, anti-diabetic, anti-cancer, and cardioprotective properties due to their nutritional profile and diverse phytochemical composition. The present study aimed to investigate the anti-diabetic potential of selected traditional rice varieties, including Annamazhagi, Karuppu kavuni, Karunkuruvai, Mappillai Samba, Milagu Samba, and Poongar. Metabolic profiling using gas chromatography–mass spectrometry (GC–MS) identified 561 compounds across the selected rice varieties. The top 20 compounds from each variety were selected based on retention time and relative abundance and pooled into a non-redundant compound dataset. These compounds were further subjected to network and pathway enrichment analysis, followed by ADMET prediction and virtual screening against peroxisome proliferator-activated receptor gamma (PPARγ; PDB ID: 3G9E), a key regulator of glucose homeostasis and insulin sensitivity. Molecular docking using PyRx, followed by refined docking with the Schrödinger Glide XP module and binding free energy analysis using Schrödinger Prime MM-GBSA, identified oleic acid (PubChem ID: 445639) as the most promising compound, with a docking score of −9.451 kcal/mol and binding free energy (ΔG_bind_) of −108.21 kcal/mol, compared to the reference antidiabetic drug pioglitazone (PubChem ID: 4829; −8.759 kcal/mol ΔG_bind_ = −96.81 kcal/mol). To further evaluate ligand–receptor stability, a 200 ns molecular dynamics simulation was performed using GROMACS with the CHARMM36 force field. Structural analyses, including root mean square deviation (RMSD), root mean square fluctuation (RMSF), radius of gyration (Rg), solvent-accessible surface area (SASA), hydrogen bond interactions, principal component analysis (PCA), free energy landscape (FEL), and Molecular Mechanics/Poisson–Boltzmann Surface Area (MM-PBSA) binding energy calculations analysis, indicated stable binding behavior of the PPARγ-oleic acid complex throughout the simulation. These findings suggest that oleic acid may act as a potential natural modulator of PPARγ, highlighting the therapeutic potential of traditional rice varieties as sources of antidiabetic bioactive compounds.

## 1 Introduction

Rice (*Oryza sativa* L.) belongs to the Poaceae family and is one of the most important staple crops after wheat. It serves as a primary food source for more than two-thirds of the world's population and is cultivated on approximately 167.2 million acres globally with an annual production of about 769.4 million tons in India alone, rice is grown on nearly 45 million hectares of land with a production of around 125 million tons (1). Rice is a rich source of carbohydrates, containing small amounts of protein and fat and provides B-complex vitamins, including thiamine, riboflavin, and niacin. A typical rice grain contains approximately 12% water, 75%−80% starch and 7% protein, along with essential minerals such as magnesium, phosphorus, and calcium, as well as trace elements including iron, zinc, copper, and manganese (2).

Traditional rice varieties, often known as heirloom or heritage types, exhibit greater nutritional richness and diversity than modern improved cultivars (3). These traditional rice varieties are characterized by lower sugar content, higher dietary fiber, and elevated levels of glutamic acid, which collectively contribute to improved glycemic control and metabolic health. They are also rich sources of essential vitamins and minerals, including niacin, thiamine, riboflavin, vitamin D, iron, calcium, and zinc (4). Beyond their superior nutrient profile, these traditional varieties possess remarkable genetic diversity, medicinal properties, unique aromatic characteristics, and enhanced resilience to environmental stresses (5). Traditional rice varieties such as Karuppu Kavuni, Mappillai Samba, Poongar, and Karunkuruvai have been reported to contain anthocyanins and other phyto-bioactive compounds exhibiting antioxidant, anti-inflammatory, anti-cancer, and anti-diabetic properties (6–8).

Diabetes mellitus (DM) is a metabolic condition defined by elevated blood glucose levels and abnormalities in metabolic regulation. It is broadly classified into two types: type 1 diabetes mellitus is characterized by inadequate insulin production and type 2 diabetes mellitus arises when the body's cells are unable to properly respond to insulin (9). T2DM is the most prevalent form and is strongly influenced by lifestyle-related risk factors such as physical inactivity, unhealthy dietary habits, obesity and advancing age, while genetic predisposition acts as a contributing rather than determining factor (10). The International Diabetes Federation estimates that 537 million individuals were affected with diabetes in 2021, projected to rise to 783 million by 2045, with nearly 90% of cases attributed to T2DM (11).

Peroxisome proliferator-activated receptors (PPARs) are ligand-dependent nuclear hormone receptors that regulate the expression of genes essential for several physiological functions, such as glucose and lipid balance, inflammatory responses, and cell differentiation (12, 13). This receptor occurs in three isoforms, which include PPARα, PPARγ, and PPARδ, each with a distinct metabolic role (14). While all three subtypes differ in ligand preference, activity and tissue expression, PPARγ has been the most extensively studied due to its crucial role in metabolic diseases (15). It is predominantly expressed in cells with high triacylglyceride (TAG) content, particularly in white and brown adipose tissues and is also present in immune cells such as monocytes and macrophages (16). PPARγ plays a vital role in several physiological processes by enhancing insulin sensitivity through the upregulation of glucose transporter type-4 (GLUT-4), promoting lipid storage in adipose tissue and exerting anti-inflammatory effects that reduce insulin resistance, thereby making it an important therapeutic target for T2DM and related metabolic disorders (17). PPARγ agonists are insulin-sensitizing drugs that improve glucose metabolism by regulating genes involved in glucose uptake, adipocyte differentiation and lipid storage, thereby enhancing insulin sensitivity in adipose tissue, liver and skeletal muscle (17). Pioglitazone, a thiazolidinedione and PPARγ agonist, is used to improve glycemic control in T2DM. However, clinical studies, including the PROactive trial, have reported adverse effects such as an increased risk of bladder cancer, leading to safety concerns and restricted use in certain countries (18, 19). Since the approval of pioglitazone in 1999, no new drugs targeting insulin resistance have gained regulatory approval. This is largely due to the emergence of undesirable side effects during clinical trials (20). These limitations emphasize the need to explore safer natural compounds that can effectively modulate PPARγ activity.

Traditional rice varieties have gained increasing attention due to their rich nutritional composition and presence of diverse bioactive metabolites. Several studies have reported the anti-diabetic potential of traditional rice varieties, mainly through nutritional analysis, glycemic index evaluation, antioxidant activity, and inhibition of digestive enzymes such as α-amylase and α-glucosidase (21, 22). These studies clearly demonstrate that traditional rice consumption can reduce postprandial hyperglycemia. However, they primarily focus on enzyme inhibition and short-term glucose regulation. Despite the clinical importance of PPARγ as a therapeutic target in T2DM, existing literature on PPARγ agonists has largely focused on synthetic drugs and bioactive compounds derived from medicinal plants (23). Although GC–MS-based and nutraceutical profiling studies have reported the presence of bioactive metabolites in traditional rice (1, 5, 24), systematic evaluation of their bioactive compounds against PPARγ remains limited. The present study aims to investigate the anti-diabetic potential of selected traditional rice varieties, such as Annamazhagi, Karuppu Kavuni, Karunkuruvai, Mappillai Samba, Milagu Samba, and Poongar, by identifying GC-MS derived bioactive compounds and evaluating their interactions with PPARγ through molecular docking and molecular dynamics simulations in comparison to the standard drug pioglitazone.

## 2 Materials and methodology

### 2.1 Sample collection

Six traditional rice varieties, namely Annamazhagi, Karunkuruvai, Karuppu kavuni, Mappillai Samba, Milagu Samba, and Poongar were selected, which are majorly cultivated in the Cauvery delta regions of Tamil Nadu. Mature seeds were collected during the harvesting season directly from local farmers and the samples were cleaned, shade-dried and stored in airtight containers at room temperature until further analysis.

### 2.2 Sample preparation and GCMS analysis

A finely powdered sample (300 mg) was extracted by adding 1 mL of 100% HPLC-grade methanol in a 2 mL centrifuge tube. The mixture was subjected to vortex mixing at 2,000 rpm for 10 min, followed by sonication at 30–37 °C for 30 min. After centrifugation at 7,000 rpm for 10 min, the supernatant was collected and evaporated to dryness using a vacuum concentrator at 45 °C for 1 h. The dried extract was then derivatized by adding 70 μL of methoxyamine HCl in pyridine and incubating in a water bath at 90 rpm for 3 h. This was followed by the addition of 80 μL of MSTFA, with further incubation at 37 °C for 30 min. The mixture was centrifuged at 10,000 rpm for 10 min and the resulting supernatant was transferred into a sealed glass vial and stored at 4 °C for further GC-MS analysis.

The derivatized samples were analyzed using a Shimadzu triple quadrupole GCMS-T NX Gas Chromatograph–Mass Spectrometer (GC-MS). One microliter aliquot was injected in split mode (split ratio 1:20) into the SH-Rxi-5Sil MS column at an injector temperature of 280 °C. The column temperature was programmed to start at 70 °C and held for 1 min, then increased to 320 °C and held for 10 min. The mass spectrometer was operated with an ion source temperature of 230 °C, interface temperature of 280 °C, solvent cut time of 5.0 min and a mass range from 50 m/z to 650 m/z. The GCMS analysis was carried out at the Department of Plant Molecular Biology and Bioinformatics, Centre for Plant Molecular Biology and Biotechnology, Tamil Nadu Agricultural University, Coimbatore. Compound identification was performed by comparing mass spectral fragmentation patterns against the NIST (National Institute of Standards and Technology) and Wiley reference libraries.

### 2.3 Similarity index analysis

The detected compounds were screened and the top 20 compounds from each rice variety were selected based on area percentage (relative abundance) and retention time (compound identification). The chemical structures of the selected compounds were retrieved in SMILES format from the PubChem database (https://pubchem.ncbi.nlm.nih.gov/). Structural similarity with the reference drug pioglitazone was evaluated using the similarity workbench in ChemMine Tools (https://chemminetools.ucr.edu/). It was calculated using the tanimoto coefficient based on Atom Pair (AP) and Maximum Common Substructure (MCS) methods (25).

### 2.4 Network and pathway enrichment analysis

The top 20 compounds from each variety were pooled and duplicate entries across the six varieties were removed to obtain a non-redundant compound set. The resulting compounds were subsequently subjected to network analysis using Cytoscape version 3.10.3 (https://cytoscape.org/) and pathway enrichment analysis using MetaboAnalyst version 6.0 (https://www.metaboanalyst.ca/) with default parameters. The compounds were mapped to metabolic pathways using the Kyoto Encyclopedia of Genes and Genomes (KEGG) database, with *Arabidopsis thaliana* used as the reference organism for pathway analysis.

### 2.5 ADMET prediction

ADMET prediction was performed using ADMETlab 3.0 (https://admetlab3.scbdd.com/). To assess the compliance of compounds with desirable ADMET (Absorption, Distribution, Metabolism, Excretion, and Toxicity) characteristics, numerical values were assigned according to empirical threshold criteria. Compounds with prediction scores between 0.0 and 0.3 were considered to exhibit excellent ADMET properties (0). Scores within the range of 0.3 to 0.7 were classified as moderate [2], while those between 0.7 and 1.0 were categorized as poor [1] (26).

### 2.6 Molecular docking analysis

#### 2.6.1 Ligand preparation

The three-dimensional (3D) structures of the compounds were retrieved from the PubChem database (https://pubchem.ncbi.nlm.nih.gov/). Pioglitazone was included as the reference drug molecule to evaluate its binding affinity toward the PPARγ receptor. All compounds, together with the reference drug, were converted from SDF to PDBQT format using the PyRx tool for docking.

#### 2.6.2 Protein preparation

The crystal structure of the PPARγ receptor (PDB ID: 3G9E) (27), which contains the co-crystallized ligand aleglitazar, was obtained from the Protein Data Bank (https://www.rcsb.org/) and prepared for docking analysis. All crystallographic water molecules and heteroatoms were removed to prevent unwanted interactions within the ligand-binding site. Polar hydrogens were added to represent the protonation state of the protein appropriately and to maintain proper hydrogen-bonding geometry. These preparation steps were performed using PyRx (https://pyrx.sourceforge.io/), after which the prepared structure was converted from PDB to PDBQT format.

#### 2.6.3 Binding site identification

The binding site of the PPARγ receptor was identified using the co-crystallized ligand aleglitazar present in the 3G9E crystal structure. The amino acids located within the ligand-binding pocket were visualized using PyMOL (https://www.pymol.org/) and the residues surrounding the native ligand were selected as the binding site. The identified binding site residues include Arg 280, Ile 281, Phe 282, Gly 284, Cys 285, Gln 286, Arg 288, Ser 289, His 323, Leu 330, Val 339, Leu 340, Ile 341, Met 348, Leu 353, Phe 363, Met 364, Lys 367, His 449, and Tyr 473.

#### 2.6.4 Virtual screening using PyRx

Virtual screening was performed using PyRx, which integrates AutoDock Vina for docking analysis (28). The prepared ligand library, along with the reference drug pioglitazone, was imported into the PyRx workspace in PDBQT format. The refined PPARγ receptor was loaded as the macromolecule for docking. A grid box was generated around the active site with coordinates center_x = 3.9524, center_y = 25.5320, and center_z = 19.8797 to encompass the ligand-binding pocket. All ligands were docked using AutoDock Vina and the binding affinity values (kcal/mol) were obtained.

#### 2.6.5 Refined docking using Schrödinger

To further validate the docking results, the lowest binding affinity exhibiting compound was identified from the PyRx analysis, along with the reference drug pioglitazone. Refined docking of the identified compounds and the protein target was performed using the Schrödinger Glide XP module. In Glide XP (Extra Precision) (29) docking, the ligand was treated as flexible while the protein was maintained as a rigid structure and the scoring was performed using the OPLS3e force field. Binding free energy calculations for all 53 compounds were subsequently performed using the Schrödinger Prime MM-GBSA module (30).

### 2.7 Molecular dynamics simulation

Molecular dynamics (MD) simulations were performed for three systems: the apo form of PPARγ, the PPARγ-ligand complex with the lowest binding affinity obtained from docking and the PPARγ-pioglitazone complex, which served as a reference drug. The simulations were carried out using GROMACS 2024.4 (https://www.gromacs.org/) with the CHARMM36 force field. The ligand structures were prepared using Avogadro (https://sourceforge.net/projects/avogadro/) and ligand topology files were generated using the CGenFF server (https://cgenff.com/). Each system was solvated in a SPC water box and appropriate counter ions were added to neutralize the system. Energy minimization was performed to remove steric clashes, followed by equilibration under NVT and NPT ensembles to stabilize the temperature and pressure of the system. After equilibration, a 200 ns production MD simulation was conducted for each system. Post-simulation analyses, including root mean square deviation (RMSD), root mean square fluctuation (RMSF), radius of gyration (Rg), solvent-accessible surface area (SASA), and hydrogen bond interactions, were performed to evaluate the structural stability, compactness, flexibility, and interaction stability of the apo protein and the PPARγ complexes throughout the simulation.

### 2.8 Principal component analysis and free energy landscape

Principal component analysis (PCA) and free energy landscape (FEL) construction were performed to investigate the dominant conformational motions of the oleic acid–PPARγ complex during molecular dynamics simulations. PCA was carried out on the MD trajectory using GROMACS tools gmx covar and gmx anaeig (31). Prior to analysis, the trajectory was aligned to the reference structure to remove rotational and translational motions and the covariance matrix of backbone atomic fluctuations was constructed and diagonalized to obtain eigenvectors representing collective motions (32).

The FEL of the protein–ligand complexes were constructed by projecting the trajectories onto the first two principal components. The gmx sham tool was used to compute the free energy distribution, enabling the identification of stable and unstable conformational states. The resulting FEL was visualized using 2D contour and 3D surface plots generated in Matplotlib (31).

### 2.9 MM-PBSA analysis

The binding free energy of the PPARγ-ligand complexes was estimated using the Molecular Mechanics/Poisson–Boltzmann Surface Area (MM/PBSA) method. The analysis was performed using the gmx_MMPBSA (https://github.com/Valdes-Tresanco-MS/gmx_MMPBSA) tool based on the trajectories obtained from molecular dynamics simulations. The binding free energy (ΔG_bind_) was calculated by combining molecular mechanics energy terms, including van der Waals and electrostatic interactions, with solvation energies consisting of polar and non-polar contributions. The total binding free energy was calculated as: ΔG_bind_ = G_complex_ – (G_protein_ + G_ligand_) where G_complex_, G_protein_, and G_ligand_ represent the free energies of the complex, receptor and ligand, respectively (26).

## 3 Results

### 3.1 Metabolomic profiling of rice methanol extracts

The GC–MS profiling of traditional rice varieties revealed a total of 561 compounds, with 84 compounds identified in Annamazhagi, 94 in Karunkuruvai, 100 in Karuppukavuni, 90 in Mappillai Samba, 100 in Milagu Samba, and 93 in Poongar, as shown in Supplementary Figures S1–S6. Among the identified metabolites, the top 20 compounds from each rice variety were selected based on their area percentage (relative abundance), while retention time was used for compound identification. The metabolic profiles of these selected compounds from all six varieties, along with their similarity indices with the reference drug Pioglitazone, are presented in Supplementary Table S1. After removing duplicates, a total of 53 unique compounds were obtained from the initial list of 120 (20 per variety × 6 varieties) compounds.

Across all rice varieties, carbohydrates and fatty acids constituted the predominant classes of metabolites, followed by sugar alcohols, while sterols, organic acids, and other minor compounds were present at comparatively lower levels. Among the carbohydrates, sucrose was the most predominant compound in Annamazhagi, Karunkuruvai, Mappillai Samba, and Poongar, whereas relatively lower levels were observed in Karuppukavuni and Milagu Samba. Sugar alcohols (mannitol and maltitol) were mainly observed in Karuppukavuni, Mappillai Samba, Milagu Samba, and Poongar. Both saturated and unsaturated fatty acids were identified across all rice varieties. Palmitic acid was consistently observed in all six rice varieties, while linoleic acid [9,12-octadecadienoic acid (Z, Z)] was absent in Milagu samba. Oleic acid was found in Karuppukavuni, Milagu Samba, and Poongar. Stearic acid was found in Annamazhagi and Karunkuruvai. 1-monopalmitin was detected at lower proportions in Karunkuruvai, Mappillai Samba, and Poongar. The sterol compound cholest-5-en-3-ol was identified in all six rice varieties. Organic acids such as acetoacetic acid, hydroxybutyric acid, hydroxyvaleric acid, gulonic acid, and phosphoric acid were variably distributed among the traditional rice varieties. The similarity index analysis, based on AP and MCS tanimoto methods, indicated that the selected compounds exhibited low similarity with the reference drug pioglitazone, reflecting distinct chemical scaffolds. Linoleic acid showed the highest similarity in the AP method (0.129), while coniferyl aldehyde showed the highest similarity in the MCS method (0.266).

**Figure 1:**
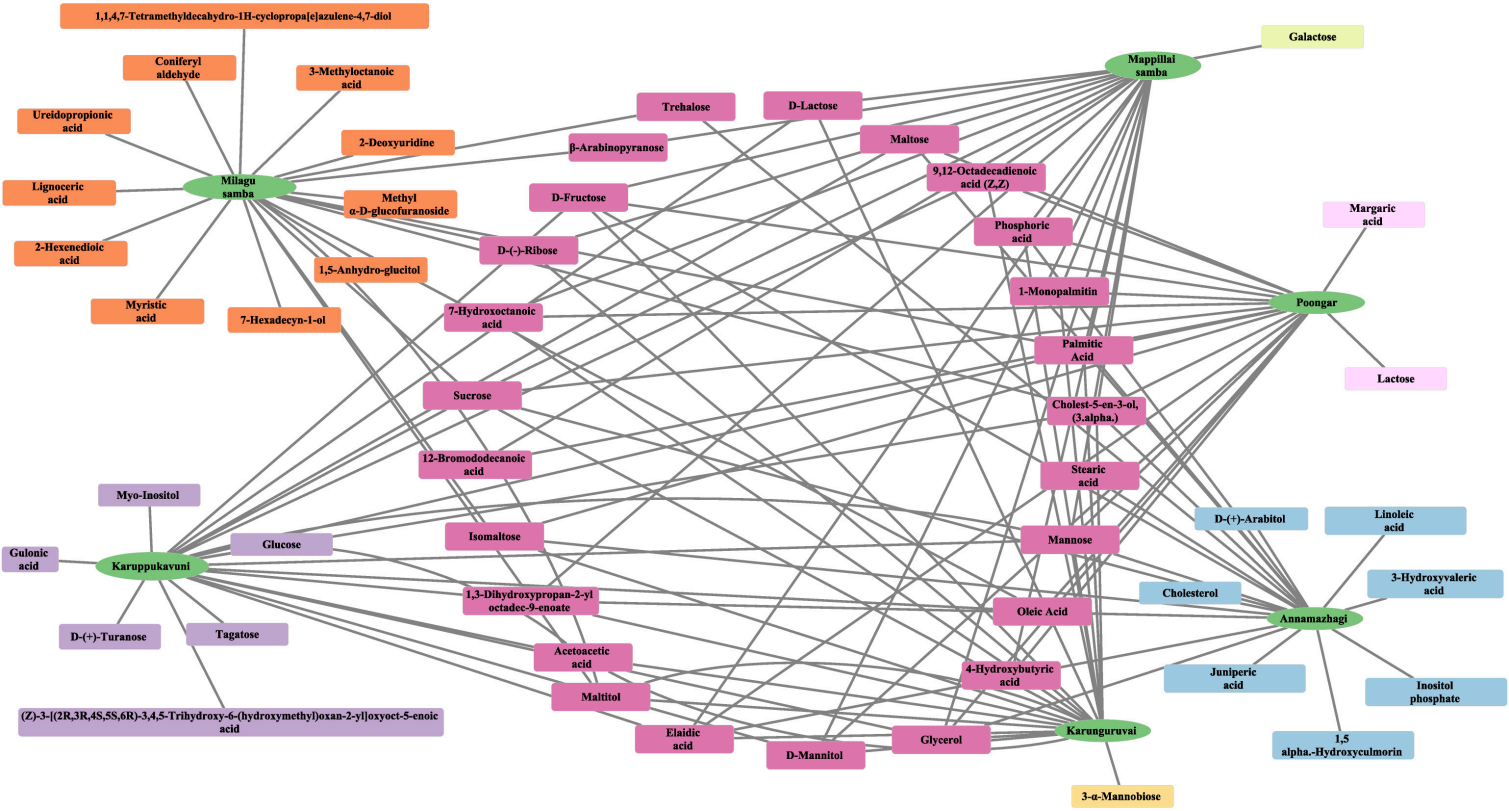
Network representation of shared and unique metabolites across traditional rice varieties.

To further visualize these compound distributions, a network-based analysis was performed using Cytoscape software (Figure 1). The resulting network illustrates the relationships between rice varieties and their associated compounds, highlighting central hub compounds that are shared across multiple varieties. It should be noted that several compounds formed variety-specific clusters, reflecting distinct metabolic profiles among individual traditional rice varieties.

### 3.2 Pathway enrichment analysis

**Figure 2:**
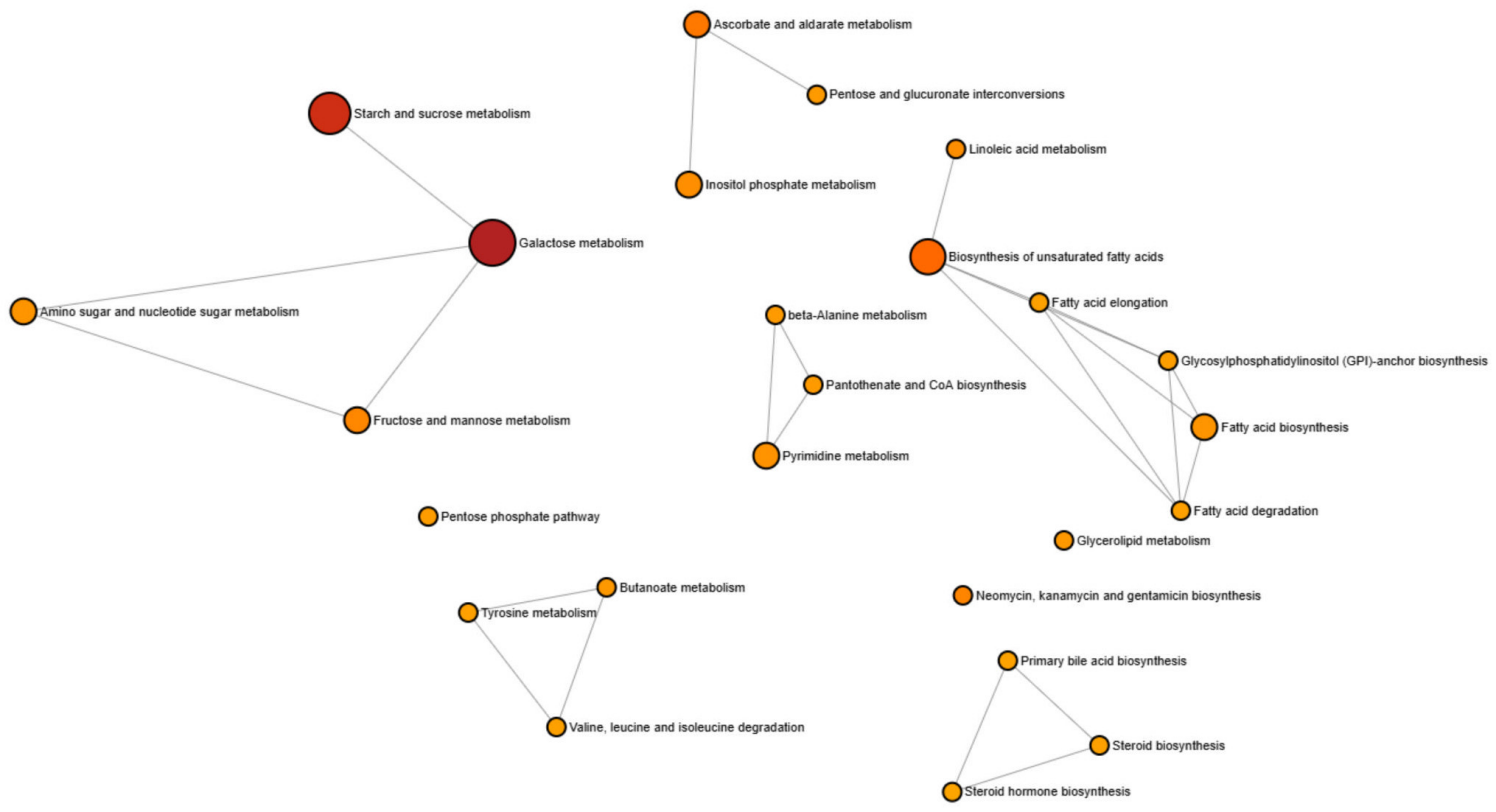
Pathway interaction network of metabolites identified from traditional rice varieties.

Pathway enrichment analysis was performed to investigate the biological processes associated with the identified compounds. The resulting pathway interaction network is shown in Figure 2, where nodes represent enriched metabolic pathways and edges indicate shared compounds between pathways. Larger nodes correspond to pathways involving a greater number of compounds. The analysis revealed that pathways related to carbohydrate metabolism, including galactose metabolism, starch and sucrose metabolism, were prominently enriched. In addition, several lipid-related pathways such as fatty acid biosynthesis, biosynthesis of unsaturated fatty acids, fatty acid elongation, fatty acid degradation, linoleic acid metabolism, and glycerolipid metabolism were also represented. Other supporting pathways included the pentose phosphate pathway, amino acid metabolism, steroid biosynthesis, and primary bile acid biosynthesis. These results indicate that the identified compounds are mainly involved in central metabolic processes related to carbohydrate, lipid, and amino acid metabolism.

### 3.3 ADMET prediction

**Figure 3:**
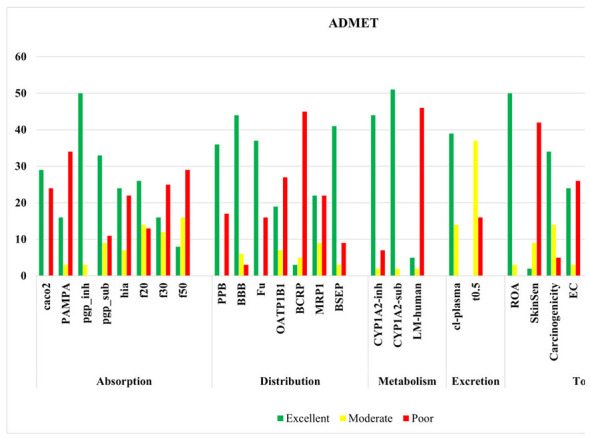
ADMET analysis of GC–MS-identified compounds from traditional rice.

ADMET prediction was performed on the 53 metabolites identified from GC–MS analysis of traditional rice varieties. The overall distribution of pharmacokinetic and toxicity parameters is presented in Figure 3. Drug-likeness assessment indicated that all compounds complied with Lipinski's rule of five, suggesting favorable oral drug-like properties. Absorption analysis showed that 29 compounds exhibited excellent Caco-2 permeability, while 16 demonstrated excellent PAMPA permeability. P-glycoprotein interaction analysis revealed 50 compounds as P-gp inhibitors and 33 as P-gp substrates. Human intestinal absorption predictions classified 24 compounds as excellent. Oral bioavailability evaluation indicated that 26, 16, and 8 compounds met the 20%, 30%, and 50% thresholds, respectively.

Distribution analysis demonstrated favorable pharmacokinetic behavior, with 36 compounds showing excellent plasma protein binding, 44 exhibiting optimal volume of distribution and 37 displaying blood–brain barrier permeability. Transporter-based assessments identified 19 compounds with favorable OATP1B1 activity, 41 with BSEP interaction and 22 with acceptable MDR1 profiles. Metabolic evaluation revealed that 44 and 51 compounds showed excellent CYP1A2 inhibitor and substrate profiles, respectively.

Excretion profiling indicated that 39 compounds exhibited favorable plasma clearance and 37 compounds showed acceptable half-life values, suggesting sustained systemic exposure. Toxicity assessment showed that 47 compounds were predicted to be non-neurotoxic, 37 were non-hematotoxic, 50 exhibited no acute oral toxicity, and 34 compounds were classified as non-carcinogenic. ADMET results indicate that a substantial proportion of the 53 compounds possess favorable pharmacokinetic properties with minimal predicted toxicity, supporting their potential for further analysis.

### 3.4 Virtual screening and molecular docking

Virtual screening of 53 metabolites, along with the reference antidiabetic drug pioglitazone (PubChem ID: 4829), was performed against the PPARγ receptor (PDB ID: 3G9E) using PyRx to identify compounds with favorable binding affinity. The binding affinity and MM-GBSA binding free energy values are presented in Supplementary Table S2. Among the screened compounds, oleic acid (PubChem ID: 445639) exhibited the most favorable interaction with the PPARγ ligand-binding site.

**Figure 4:**
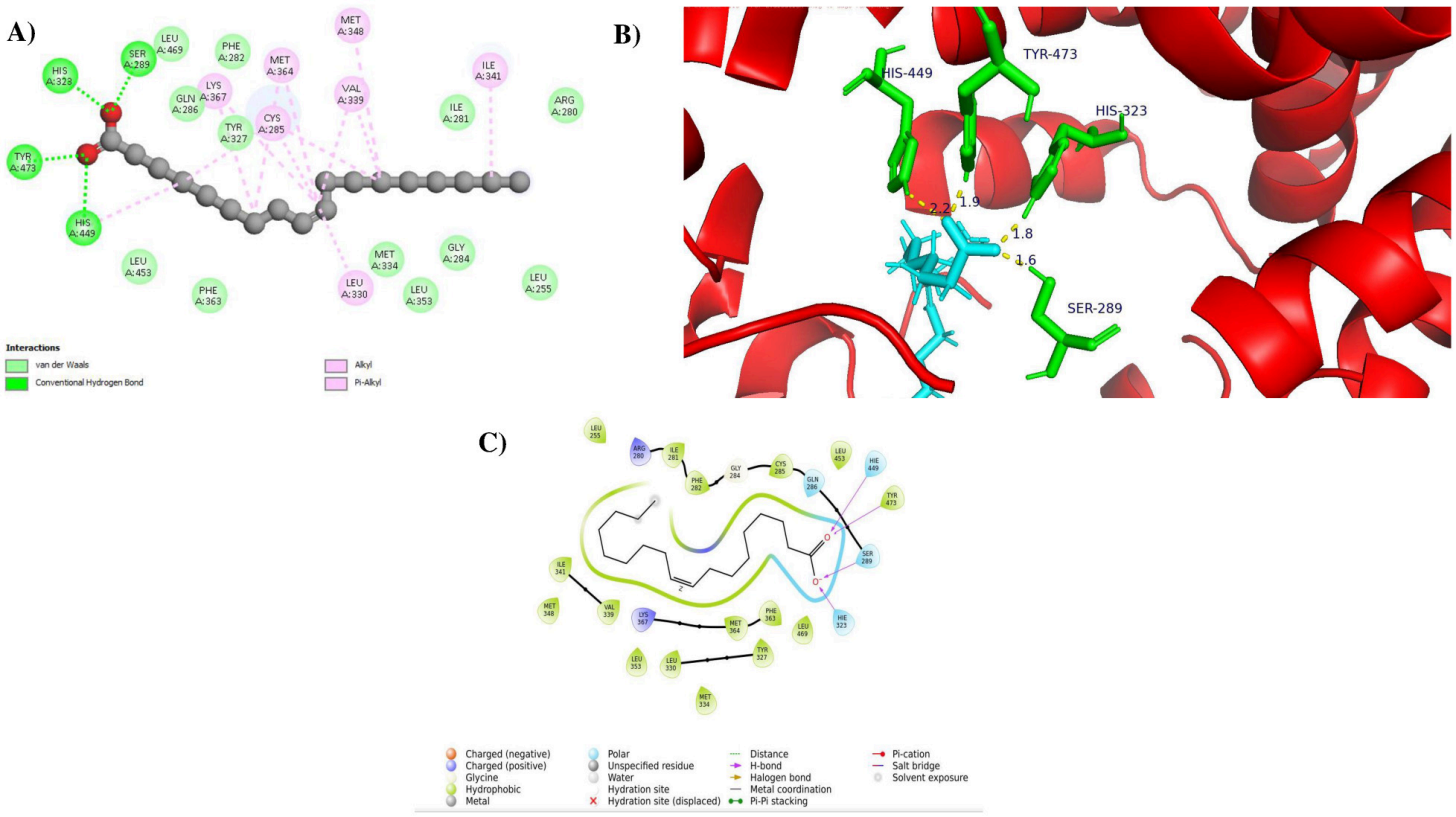
Molecular docking interactions of oleic acid with the PPARγ receptor (PDB ID: 3G9E). **(A)** Two-dimensional interaction diagram depicting the hydrogen bond interactions between oleic acid and the receptor residues, **(B)** Three-dimensional binding pose of oleic acid within the PPARγ active site, highlighting hydrogen bond contacts with key residues Ser 289, His 323, His 449, and Tyr 473, **(C)** Binding pocket visualization showing the distribution of polar and hydrophobic residues that contribute to the stabilization of oleic acid within the PPARγ receptor cavity.

As shown in Figures 4A–C, oleic acid exhibited a docking score of −9.451 kcal/mol and a favorable binding free energy of −108.21 kcal/mol, forming four conventional hydrogen bonds with key active-site residues SER A:289 (1.8 Å), HIS A:323 (1.6 Å), HIS A:449 (2.2 Å), and TYR A:473 (1.9 Å). In addition to hydrogen bonding, extensive hydrophobic interactions were observed in the form of alkyl and π-alkyl contacts with residues CYS A:285, LEU A:330, ILE A:341, VAL A:339, MET A:348, MET A:364, and LYS A:367, contributing to stable ligand accommodation within the PPARγ binding pocket. Schrödinger cavity analysis indicated that oleic acid occupies the PPARγ ligand-binding cavity effectively through a combination of polar and hydrophobic interactions.

**Figure 5:**
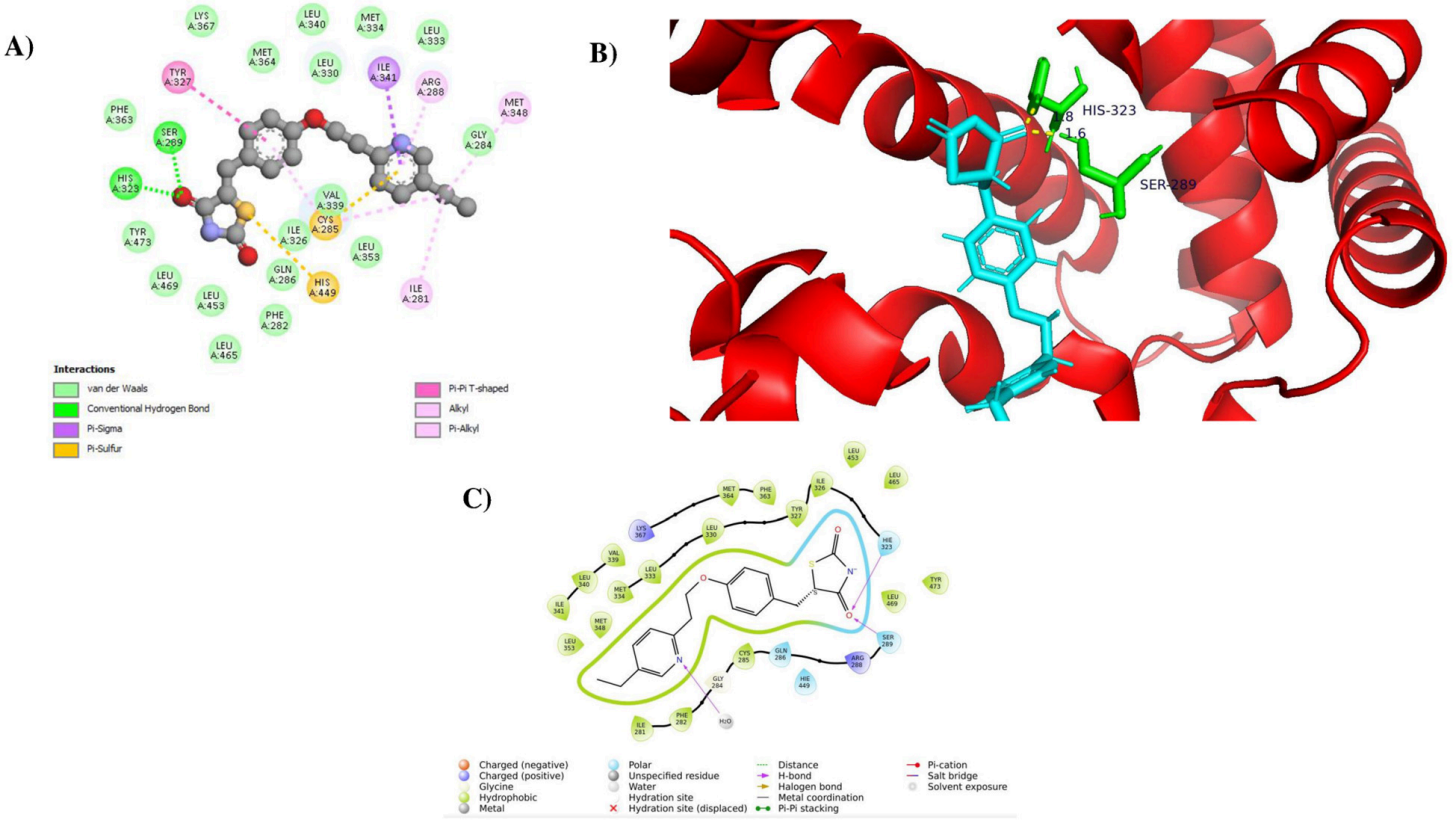
Molecular docking interactions of pioglitazone with the PPARγ receptor (PDB ID 3G9E). **(A)** Two-dimensional interaction diagram illustrating hydrogen bond and hydrophobic interactions between pioglitazone and residues within the PPARγ, **(B)** Three-dimensional binding pose of pioglitazone docked into the PPARγ active site, highlighting hydrogen bond interactions with key residues Ser 289 and His 323, **(C)** Binding pocket representation showing the surrounding polar and hydrophobic residues contributing to the stabilization of pioglitazone within the receptor cavity.

Whereas, the reference drug pioglitazone exhibited a docking score of −8.759 kcal/mol, a binding free energy of −96.81 kcal/mol, forming two conventional hydrogen bond interactions, primarily with residues SER A:289 and HIS A:323, as shown in Figures 5A–C. Pioglitazone also displayed hydrophobic interactions, including alkyl, π-alkyl, π-sigma and π-sulfur contacts with residues such as CYS A:285, ILE A:326, VAL A:339, LEU A:330, LEU A:333, MET A:348, MET A:364, PHE A:282 and ARG A:288, which support its stable binding within the canonical PPARγ ligand-binding cavity.

The docking and MM-GBSA results summarized in Table 1 indicate that oleic acid forms favorable interactions with key residues in the PPARγ ligand-binding pocket.

**Table 1:** Molecular docking and binding free energy results of oleic acid and pioglitazone.

Compound name	Docking score (kcal/mol)	MM-GBSA (kcal/mol)	Hydrogen bond interacting residues	Hydrogen bond distance
Oleic acid (445639)	−9.451	−108.21	Ser 289	1.6
			His 323	1.8
			His 449	2.2
			Tyr 473	1.9
Pioglitazone (4829)	−8.759	−96.81	Ser 289	1.6
			His 323	1.8

### 3.5 Molecular simulation

#### 3.5.1 RMSD analysis

**Figure 6:**
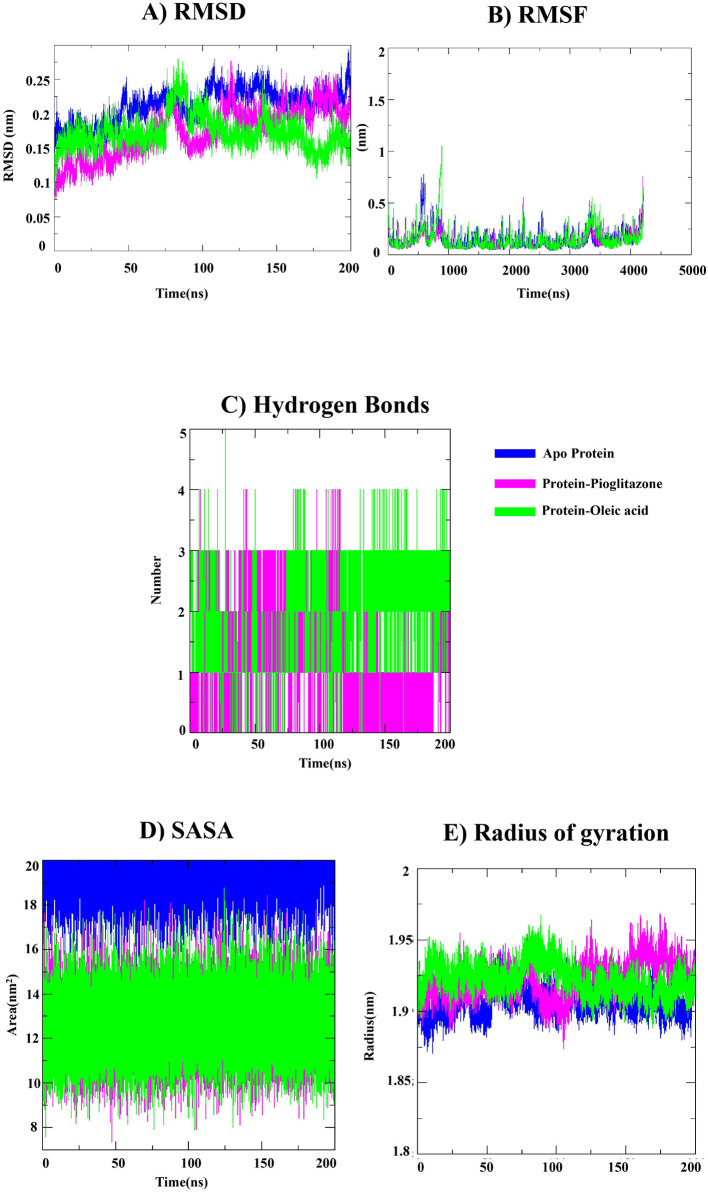
Molecular dynamics simulation analysis of PPARγ complexes. **(A)** RMSD, **(B)** RMSF, **(C)** Hydrogen bonds, **(D)** SASA, and **(E)** Radius of gyration.

Figure 6A illustrates the RMSD analysis was performed to evaluate the structural deviations of the apo PPARγ protein, the PPARγ- pioglitazone complex and the PPARγ-oleic acid complex during the 200 ns molecular dynamics simulation. The apo protein exhibited comparatively higher fluctuations, with RMSD values increasing from approximately 0.17 nm to 0.27 nm during the simulation, indicating greater conformational variability in the absence of a bound ligand. The PPARγ-pioglitazone complex showed RMSD values ranging between 0.18 and 0.24 nm after the initial equilibration phase, suggesting that the complex maintains an overall stable conformation. The PPARγ-oleic acid complex displayed RMSD values around 0.14–0.18 nm during the early stage of the simulation. A temporary increase in RMSD reaching approximately 0.26 nm was observed between 70 and 90 ns. After this period, the RMSD values fluctuated within the range of 0.15–0.20 nm, indicating stable structural behavior of the complex. The RMSD analysis demonstrates that ligand binding contributes to stabilizing the PPARγ structure, as both ligand-bound complexes show lower and more consistent RMSD values compared to the apo protein. The comparable RMSD behavior of the oleic acid and pioglitazone complexes suggests that oleic acid can maintain stable interactions with the PPARγ receptor during the simulation.

#### 3.5.2 RMSF analysis

RMSF analysis was performed to evaluate the residue-level flexibility of the PPARγ backbone (Cα atoms) during the 200 ns molecular dynamics simulation. The RMSF profiles of the apo PPARγ protein, PPARγ-pioglitazone complex, and PPARγ-oleic acid complex are presented in Figure 6B. As shown in the figure, most residues across all three systems exhibit low fluctuations, ranging from approximately 0.05–0.30 nm, indicating overall structural stability of the protein backbone during the simulation. The apo PPARγ protein (blue) displays relatively higher fluctuations at several regions, particularly around residue indices 500–800 and near the terminal regions, where RMSF values approach ranging from 0.6–0.7 nm. These fluctuations correspond to flexible loop or surface-exposed regions of the protein. In comparison, the PPARγ-pioglitazone complex shows a similar fluctuation pattern but with slightly reduced amplitude in several regions, suggesting that ligand binding helps stabilize certain segments of the protein structure. The PPARγ-oleic acid complex exhibits comparable RMSF values to the pioglitazone complex across most residues, with moderate fluctuations observed around residue indices 700–900, where RMSF values approach near to 1.0 nm. These fluctuations are mostly localized to loop regions and do not significantly affect the overall stability of the protein backbone. These results suggest that binding of oleic acid does not significantly disrupt the structural stability of PPARγ and maintains comparable backbone flexibility during the simulation.

#### 3.5.3 Hydrogen bonds

Hydrogen bond analysis was performed to evaluate the stability of intermolecular interactions between PPARγ and the bound ligands during the 200 ns molecular dynamics simulation. The number of hydrogen bonds formed between the protein and ligands throughout the simulation is shown in Figure 6C. As illustrated in the figure, the PPARγ-oleic acid complex consistently forms 1–3 hydrogen bonds during most of the simulation time, with occasional increases up to 4–5 hydrogen bonds at certain time intervals. The relatively continuous presence of hydrogen bonds indicates stable intermolecular interactions between oleic acid and residues within the PPARγ binding pocket. In comparison, the PPARγ-pioglitazone complex shows a more variable hydrogen bonding pattern. The number of hydrogen bonds fluctuates between 0 and 3, with several intervals where no hydrogen bonds are observed. These variations suggest intermittent interactions between pioglitazone and the receptor during the simulation.

The hydrogen bond analysis indicates that both complexes maintain interactions with the PPARγ receptor throughout the simulation, while the oleic acid complex exhibits relatively consistent hydrogen bonding behavior over time.

#### 3.5.4 SASA

Figure 6D illustrates the SASA of the apo PPARγ protein, PPARγ-oleic acid complex, and PPARγ-pioglitazone complex during the 200 ns molecular dynamics simulation. SASA analysis was performed to evaluate the solvent exposure of the protein under different conditions. As shown in Figure 6D, the apo PPARγ protein exhibits higher SASA values, fluctuating approximately between 16 and 19 nm^2^ throughout the simulation, indicating greater solvent exposure in the absence of a bound ligand. whereas both ligand-bound complexes display relatively lower SASA values compared to the apo protein. The PPARγ-oleic acid complex shows SASA values ranging between approximately 10–16 nm^2^, while the PPARγ-pioglitazone complex fluctuates within a similar range of 9–16 nm^2^ during the simulation. The SASA analysis indicates that the apo protein maintains higher solvent exposure, whereas ligand binding leads to reduced solvent accessibility and relatively compact structural behavior of the protein.

#### 3.5.5 Radius of gyration

Figure 6E illustrates the Rg of the apo PPARγ protein and its complexes with oleic acid and pioglitazone during the 200 ns molecular dynamics simulation. The apo protein exhibited Rg values between 1.88 and 1.91 nm, indicating stable structural compactness in the absence of ligand binding. The PPARγ-oleic acid and PPARγ-pioglitazone complexes showed slightly higher Rg values ranging from 1.90–1.96 nm, suggesting minor structural expansion upon ligand binding. Small fluctuations observed indicate that the protein structure remained stable in both apo and ligand-bound systems throughout the simulation.

### 3.6 Principal component analysis and free energy landscape

**Figure 7:**
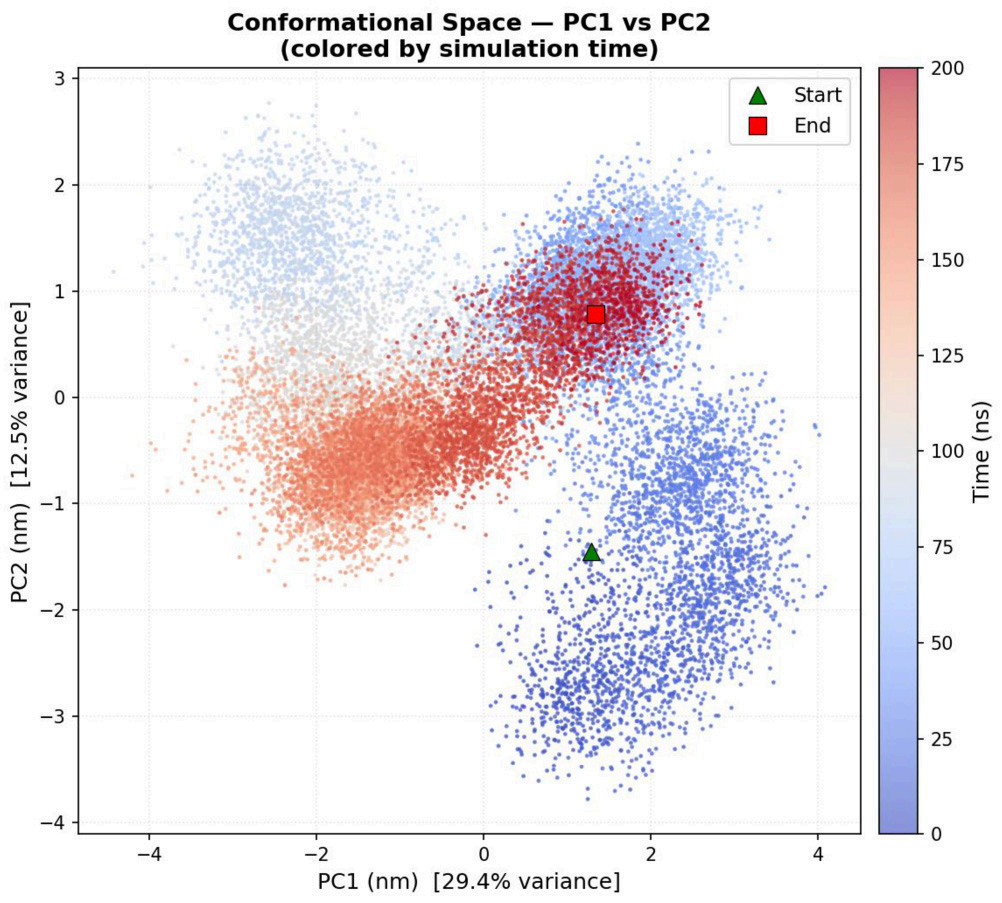
Principal Component Analysis (PCA) of the oleic acid–PPARγ complex showing conformational space along PC1 and PC2.

PCA of the PPARγ-oleic acid complex illustrates the conformational behavior of the system over time. In the PCA plot (Figure 7), the color gradient represents simulation time, progressing from blue (initial stage) to red (final stage). The distribution of points shows that the complex initially occupies a specific conformational region and gradually shifts toward other regions as the simulation progresses. The presence of distinct clusters indicates that the complex adopts multiple conformational states during the simulation and eventually reaches a more stable region toward the final phase.

**Figure 8:**
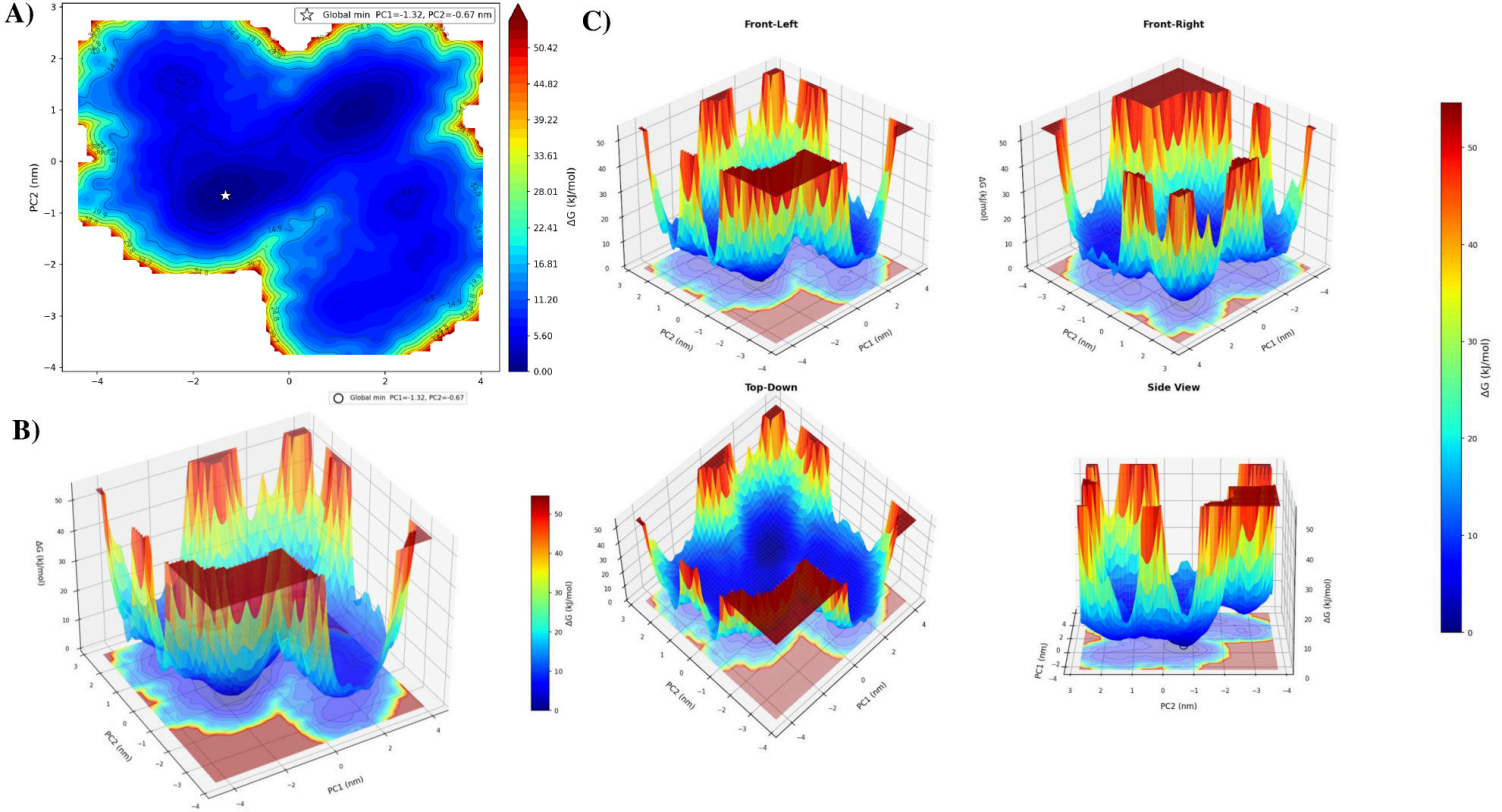
Free energy landscape (FEL) of the oleic acid–PPARγ complex derived from principal component analysis (PCA). **(A)** Two-dimensional contour plot of the FEL constructed using the first two principal components (PC1 and PC2), where the color scale represents Gibbs free energy (ΔG, kJ/mol). The global minimum energy state is indicated by a star. **(B)** Three-dimensional surface representation of the FEL showing the energy distribution across the PC1–PC2 conformational space. **(C)** Three-dimensional views of the FEL (front-left, front-right, top-down and side views) illustrating the topology of the energy landscape.

FEL further explains the stability of these conformations. The 2D contour map (Figure 8A) shows that the dark blue regions correspond to low free energy states, representing highly stable conformations of the complex. In contrast, the yellow to red regions indicate higher free energy states, which are less stable and less frequently occupied. The 3D FEL plots (Figures 8B, C) clearly illustrate deep energy basins (blue) and elevated regions (red). The deep basins represent stable conformational states, while the elevated regions indicate energy barriers that limit transitions between different conformations.

The PCA and FEL analyses indicate that the PPARγ-oleic acid complex undergoes time-dependent conformational changes and stabilizes in low-energy states, suggesting structural stability along with controlled flexibility.

### 3.7 MM-PBSA analysis

The MM/PBSA analysis was carried out to estimate the binding free energy of the PPARγ complexes with oleic acid and pioglitazone. As shown in Table 2, the calculated binding free energy (ΔG) for the PPARγ-oleic acid complex was −37.86 kcal/mol, indicating a favorable interaction between the ligand and the receptor. The PPARγ-pioglitazone complex showed a binding free energy of −27.13 kcal/mol, which also reflects stable binding within the active site. For the oleic acid complex, van der Waals (−39.9 kcal/mol) and electrostatic interactions (−33.97 kcal/mol) both contributed significantly to the binding, although these were partially opposed by the polar solvation energy (+40.93 kcal/mol). In the case of pioglitazone, van der Waals interactions (−45.4 kcal/mol) were the main contributing factor, while electrostatic interactions (−14.18 kcal/mol) were comparatively lower. The polar solvation energy (+37.15 kcal/mol) was observed to be unfavorable but was balanced by non-polar contributions. The solvation energy components in both complexes suggest that the unfavorable polar contribution is compensated to some extent by favorable non-polar interactions. The standard deviation values indicate that the pioglitazone complex shows lower fluctuations during the simulation, whereas the oleic acid complex exhibits relatively higher variability, suggesting some differences in dynamic behavior.

**Table 2:** Binding free energy (ΔG) values obtained from MM-PBSA analysis of PPARγ complexes.

PPARγ complexes	Frames	VDWAALS	EEL	EPB	ENPOLAR	GGAS	GSOLV	Total
PPARy-oleic acid	Average	−39.9	−33.97	40.93	−4.91	−73.87	36.02	−37.86
	SD	3.29	15.42	10.41	0.13	15.01	10.39	8.65
PPARy-pioglitazone	Average	−45.4	−14.18	37.15	−4.71	−59.58	32.45	−27.13
	SD	2.51	4.92	3.9	0.12	5.37	3.87	3.84

These results indicate that both complexes form stable interactions with PPARγ, with differences in their energy contributions and binding behavior observed during the simulation.

## 4 Discussion

Traditional rice varieties are widely recognized for their therapeutic potential, as they contain a diverse range of bioactive compounds compared to modern rice varieties. These compounds exhibit anti-inflammatory, anti-diabetic, anti-microbial and anti-tumor activities (33). The present study of GCMS profiling revealed a diverse range of metabolites across six traditional rice varieties, with carbohydrates, fatty acids, and sugar alcohols constituting the predominant metabolite classes. Sugar alcohols such as mannitol and maltitol have been reported to exhibit antioxidant activity and reduced postprandial glycemic and insulinemic responses, respectively (34, 35). Fatty acids constituted another major metabolite class identified in the present study. Saturated fatty acids such as palmitic acid have been reported to possess anti-inflammatory, antioxidant, anticancer and immune-enhancing properties (36). In contrast, unsaturated fatty acids, including linoleic and oleic acids, are known to exert antioxidant, anti-inflammatory and antimicrobial activities (37, 38). 1-monopalmitin has been reported to exhibit *in vitro* antitumor activity against non-small cell lung cancer through modulation of the PI3K/Akt signaling pathway (39). In addition, sterol compounds such as cholest-5-en-3-ol have been associated with antioxidant activity. The diverse metabolite profile observed in traditional rice varieties highlights their potential as functional foods with metabolic and therapeutic relevance (40, 41).

Molecular docking is a widely used computational approach in drug discovery for predicting protein–ligand interactions. When combined with virtual screening, it enables the rapid and efficient identification of potential drug candidates (42). Our molecular docking results revealed that oleic acid exhibited a favorable binding against the PPARγ receptor with key residues of Ser 289, His 323, His 449, and Tyr 473, which are reported to be important for the trans-activation activity of PPARγ (43, 44). Furthermore, refined docking performed using the Schrödinger Glide (XP) protocol showed a higher binding affinity of oleic acid toward PPARγ, with a docking score of −9.451 kcal/mol and a binding free energy of −108.21 kcal/mol, indicating a stable interaction within the ligand-binding domain. Consistent with these findings, previous docking studies employing the same receptor structure (PDB ID: 3G9E) reported binding affinities ranging from −6.7 to −8.7 kcal/mol for natural fatty acids and phytochemicals, including diosgenin, docosahexaenoic acid, docosapentaenoic acid, and methyl eicosatrienoate, which also interacted with conserved residues such as Ser 289, His 323, His 449, Tyr 473 (26). GC–MS profiling of the top 20 metabolites for each rice variety revealed the presence of oleic acid in Karuppu Kavuni, Milagu Samba, and Poongar. However, when all compounds detected by GC–MS were considered, oleic acid was present in four of the six rice varieties and absent only in Mappillai Samba and Karunkuruvai, suggesting a variety-dependent distribution rather than a uniform occurrence across all samples.

Oleic acid, a monounsaturated fatty acid abundant in dietary sources, has been consistently associated with improved insulin sensitivity and metabolic health (45). Recent clinical evidence indicates that higher plasma oleic acid levels are associated with a reduced risk of diabetic retinopathy in patients with type 2 diabetes, supporting a protective metabolic role of oleic acid in human diabetes (46). Miklankova et al. (47) reported that the oleic acid enhances insulin sensitivity by modulating lipid metabolism and inflammatory pathways prediabetic rat models. It elevates adiponectin and FADS1 expression in adipose tissue, decreases proinflammatory metabolites such as 20-HETE and reduces lipotoxic diacylglycerol (DAG), thereby enhancing insulin signaling and attenuating chronic inflammation. Vassiliou et al. (48) showed that oleic acid significantly reverses the inhibitory effect of the pro-inflammatory cytokine TNF-α on insulin production in glucose-responsive INS-1 pancreatic β-cells without inducing apoptosis. Further, oleic acid treatment promoted PPARγ nuclear translocation and enhanced binding to PPAR-responsive elements (PPREs), indicating activation of PPARγ-dependent transcriptional pathways that counteract inflammatory signaling. Consistent with these findings, *in vivo* studies showed that oleic-acid-enriched diets significantly reduced fasting blood glucose levels in type 2 diabetic mouse models. Rehman et al. (49) reported that oleic acid exhibits potent anti-diabetic properties through multiple molecular pathways, including enhancement of mitochondrial function via SIRT1-PGC1α activation, promotion of anti-inflammatory M2 macrophages and preservation of pancreatic β-cell function, positioning it as a promising therapeutic candidate for insulin resistance and type 2 diabetes mellitus.

Consistent with these biological observations, the present molecular docking and molecular dynamics simulation results shows that oleic acid has a higher probability to bind within the PPARγ ligand-binding domain and maintains dynamic stability throughout the simulation period. Taken together, the GC–MS profiling, molecular docking and molecular dynamics simulations suggest that oleic acid observed in traditional rice varieties may have antidiabetic potential through modulation of PPARγ-dependent signaling pathways.

## 5 Conclusion

The rising global burden of diabetes presents substantial health challenges, highlighting the need for safer and more effective therapeutic strategies. Traditional rice varieties, characterized by their rich nutritional and bioactive composition, may represent a valuable source of compounds with relevance to metabolic regulation. GC–MS profiling showed that oleic acid was observed in four of the rice varieties, indicating that its presence varies among different varieties. Computational analyses indicated that several compounds identified from traditional rice possess favorable pharmacokinetic properties. Among these, oleic acid showed strong and stable interactions with the PPARγ receptor, involving key residues associated with receptor activation and insulin sensitivity. The stability of the PPARγ-oleic acid complex further supports its potential role in modulating pathways relevant to type 2 diabetes. These findings suggest that bioactive compounds derived from traditional rice may contribute to antidiabetic activity and could have therapeutic relevance in the management of T2DM. It is also important to note that the metabolites identified through GC–MS depend on the extraction method used for metabolic profiling. Therefore, the detected compounds may vary depending on the protocol. Since the present findings are limited to *in silico* analyses, experimental validation through *in vitro* and *in vivo* studies is essential to confirm their antidiabetic efficacy and therapeutic relevance in the management of T2DM.

## Data Availability

The original contributions presented in the study are included in the article/Supplementary material, further inquiries can be directed to the corresponding author.

## Supplementary material

Supplementary Figure S1 GC–MS chromatogram of Annamazhagi.

Supplementary Figure S2 GC–MS chromatogram of Karunkurvai.

Supplementary Figure S3 GC–MS chromatogram of Karuppu Kavuni.

Supplementary Figure S4 GC–MS chromatogram of Mappillai Samba.

Supplementary Figure S5 GC–MS chromatogram of Milagu Samba.

Supplementary Figure S6 GC–MS chromatogram of Poongar.

Supplementary Table S1 GC–MS analysis of the top 20 compounds identified from traditional rice varieties.

Supplementary Table S2 Molecular docking results of 53 compounds, along with the reference drug pioglitazone.
