# Supplementary figures and images for: GC–MS metabolomic profiling and PPARγ-targeted in silico approaches for identifying a potential anti-diabetic compound from traditional rice varieties
Source: Front Nutr. 2026 May 5;13:1800615. doi: 10.3389/fnut.2026.1800615 (PMC13184374; doi:10.3389/fnut.2026.1800615)

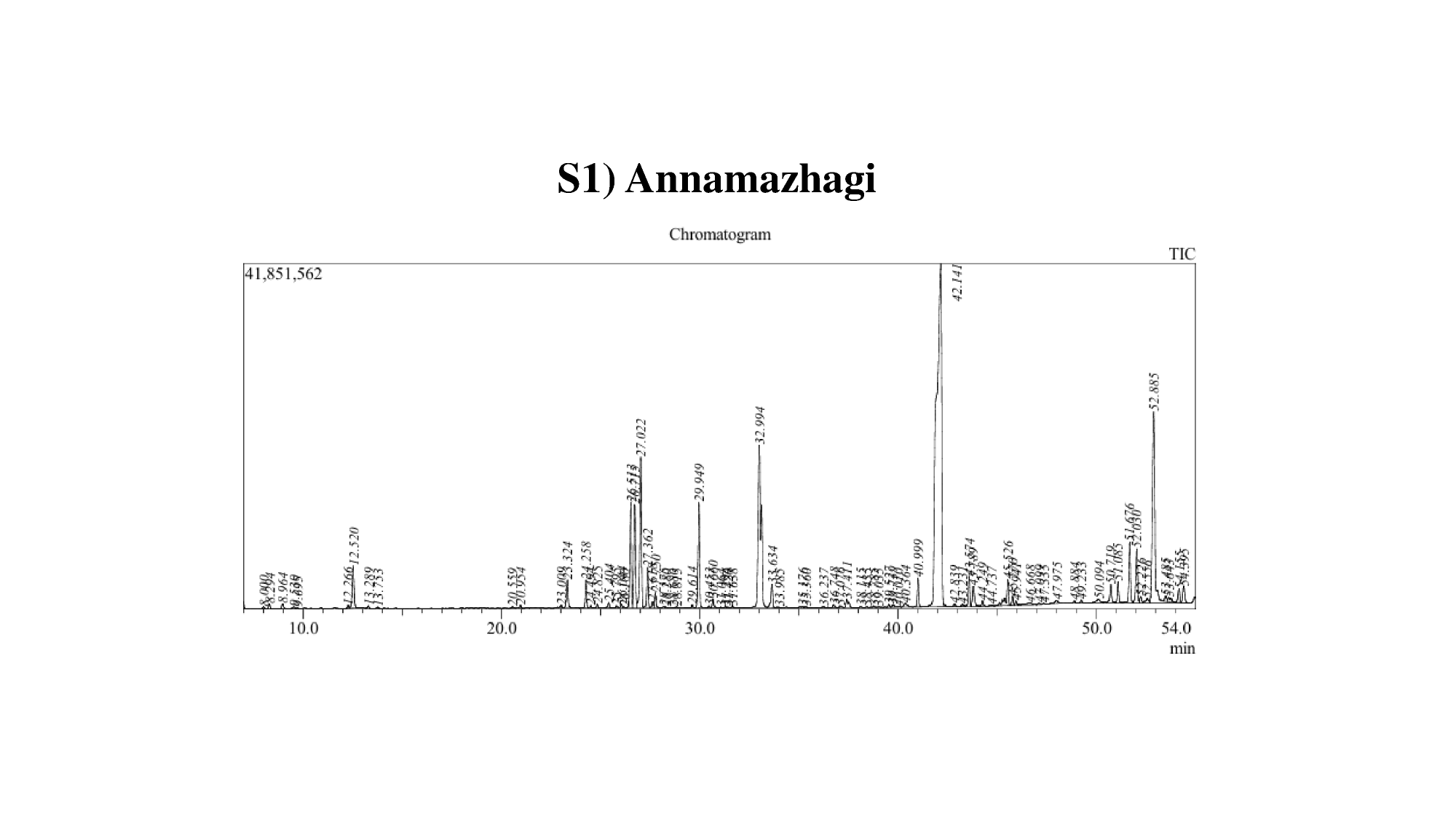

Supplement: Supplementary Figure S1 — GC–MS chromatogram of Annamazhagi. [file Image_1.tiff]

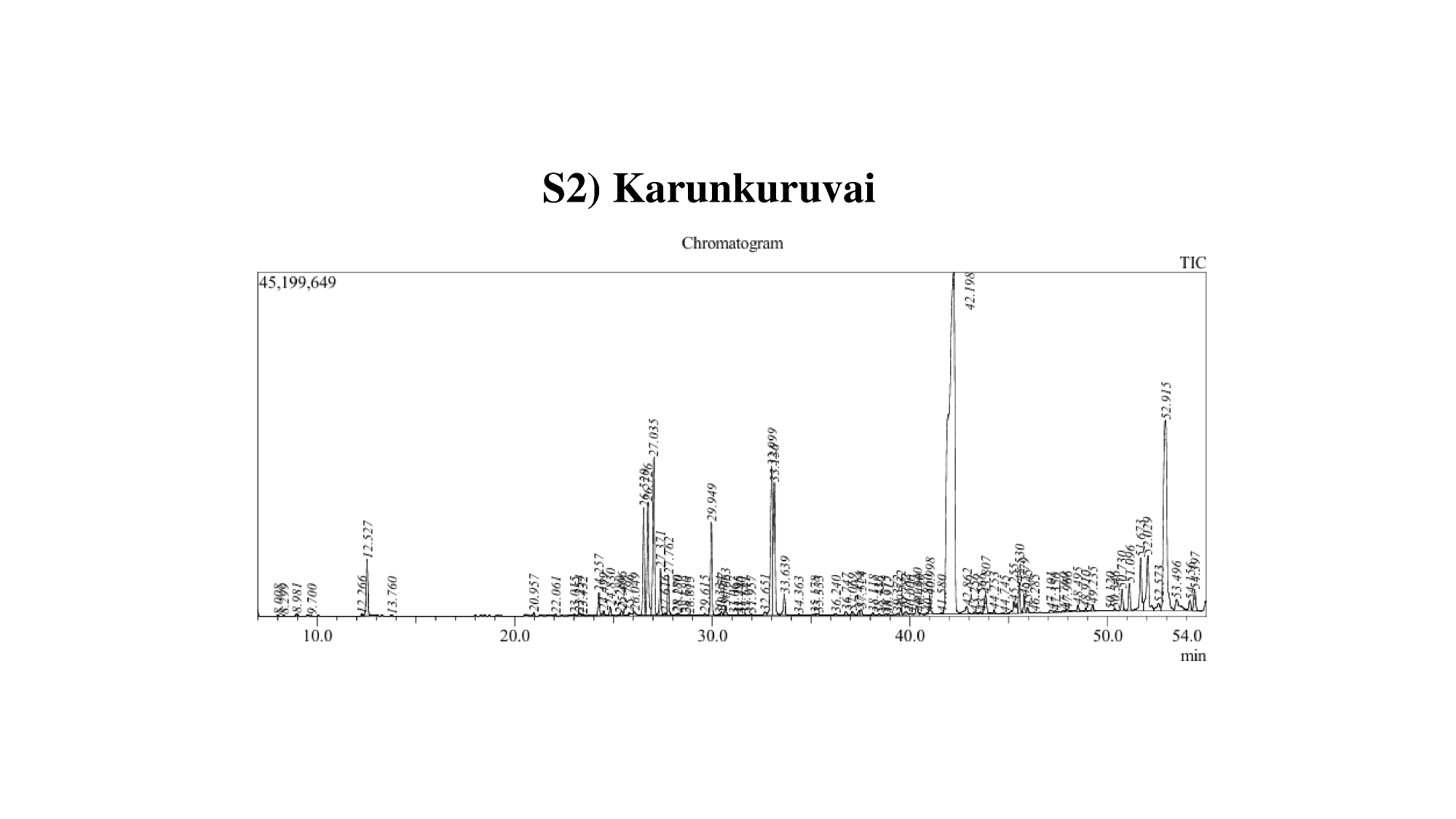

Supplement: Supplementary Figure S2 — GC–MS chromatogram of Karunkurvai. [file Image_2.tiff]

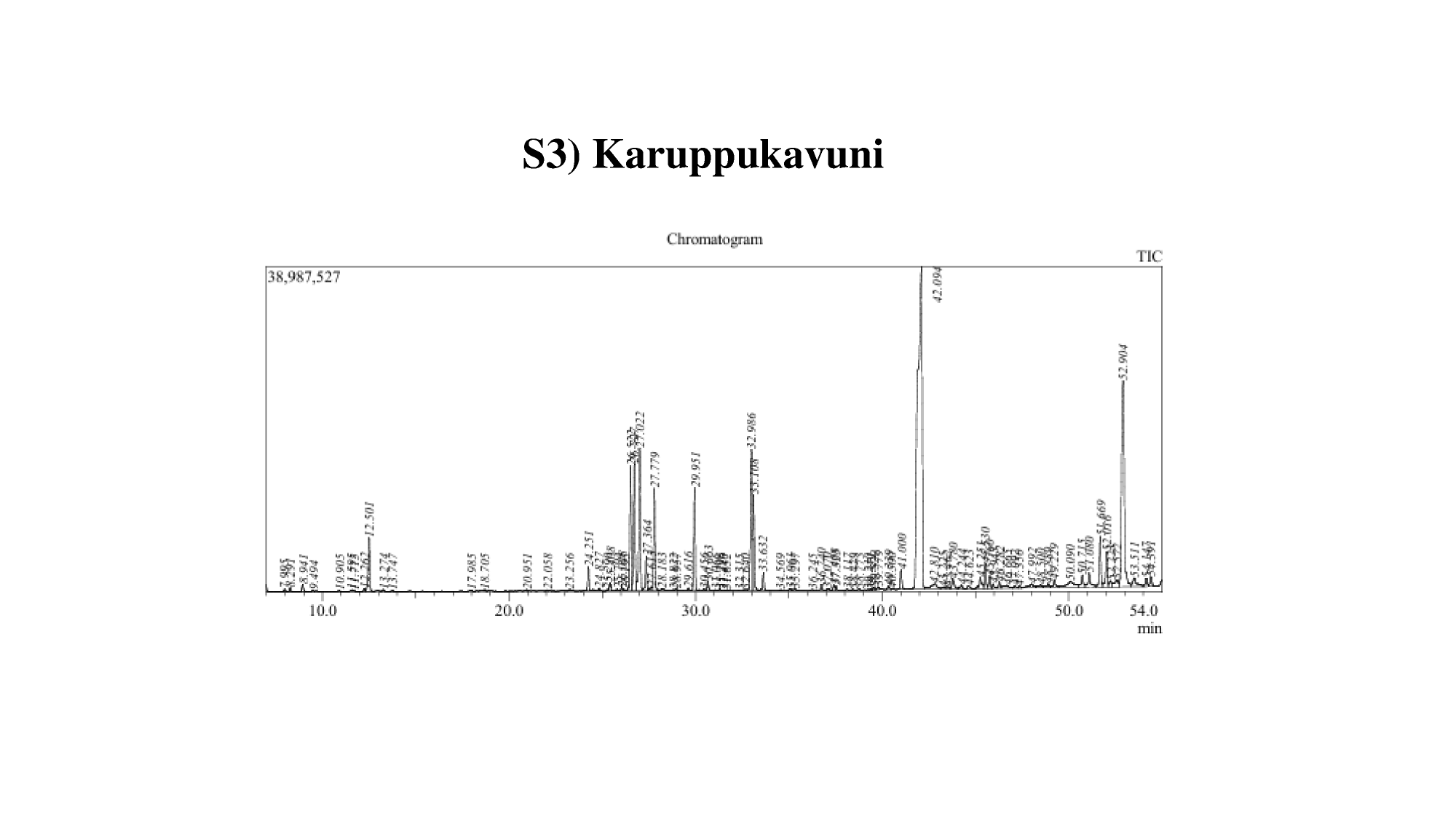

Supplement: Supplementary Figure S3 — GC–MS chromatogram of Karuppu Kavuni. [file Image_3.tiff]

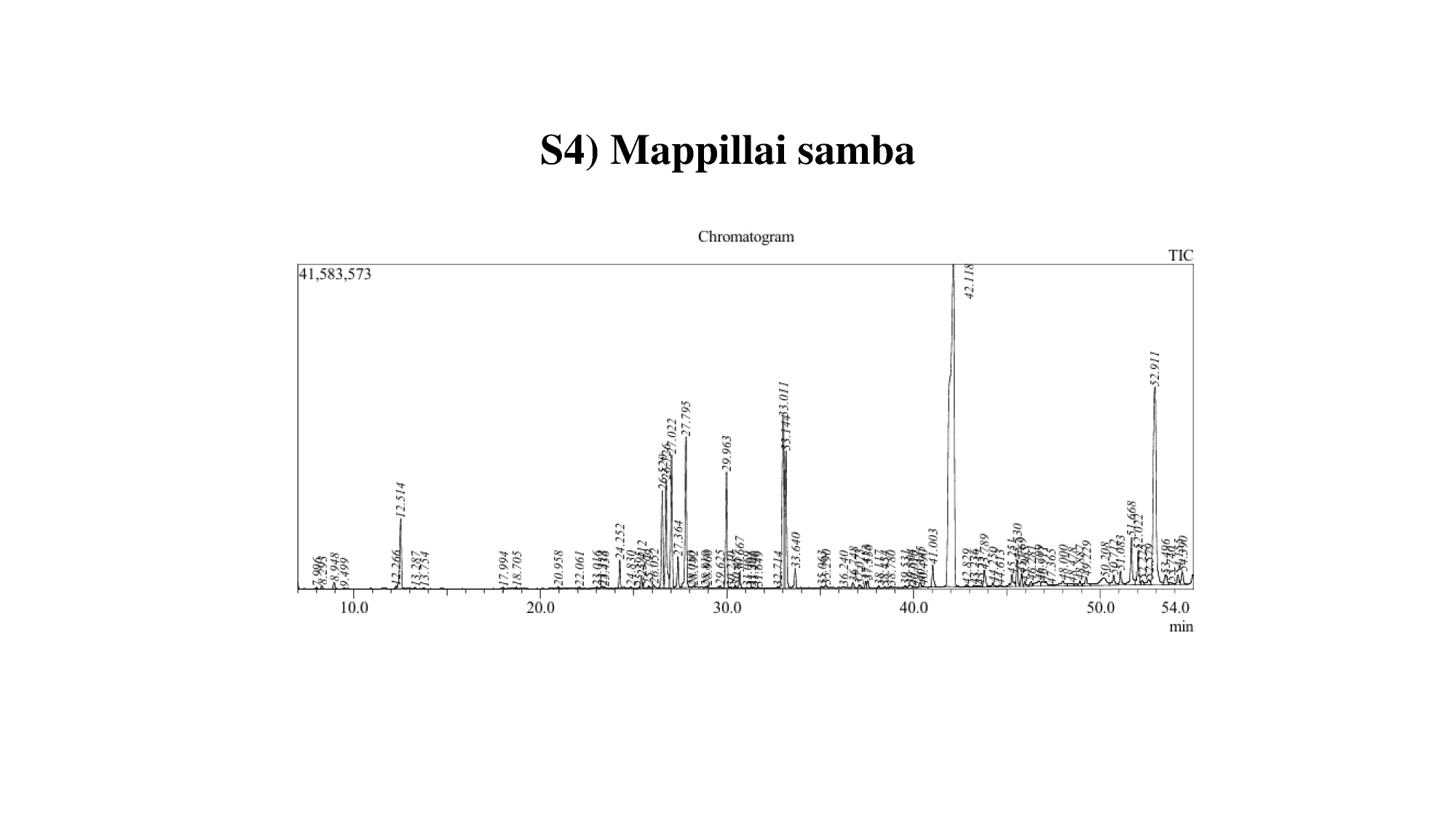

Supplement: Supplementary Figure S4 — GC–MS chromatogram of Mappillai Samba. [file Image_4.tiff]

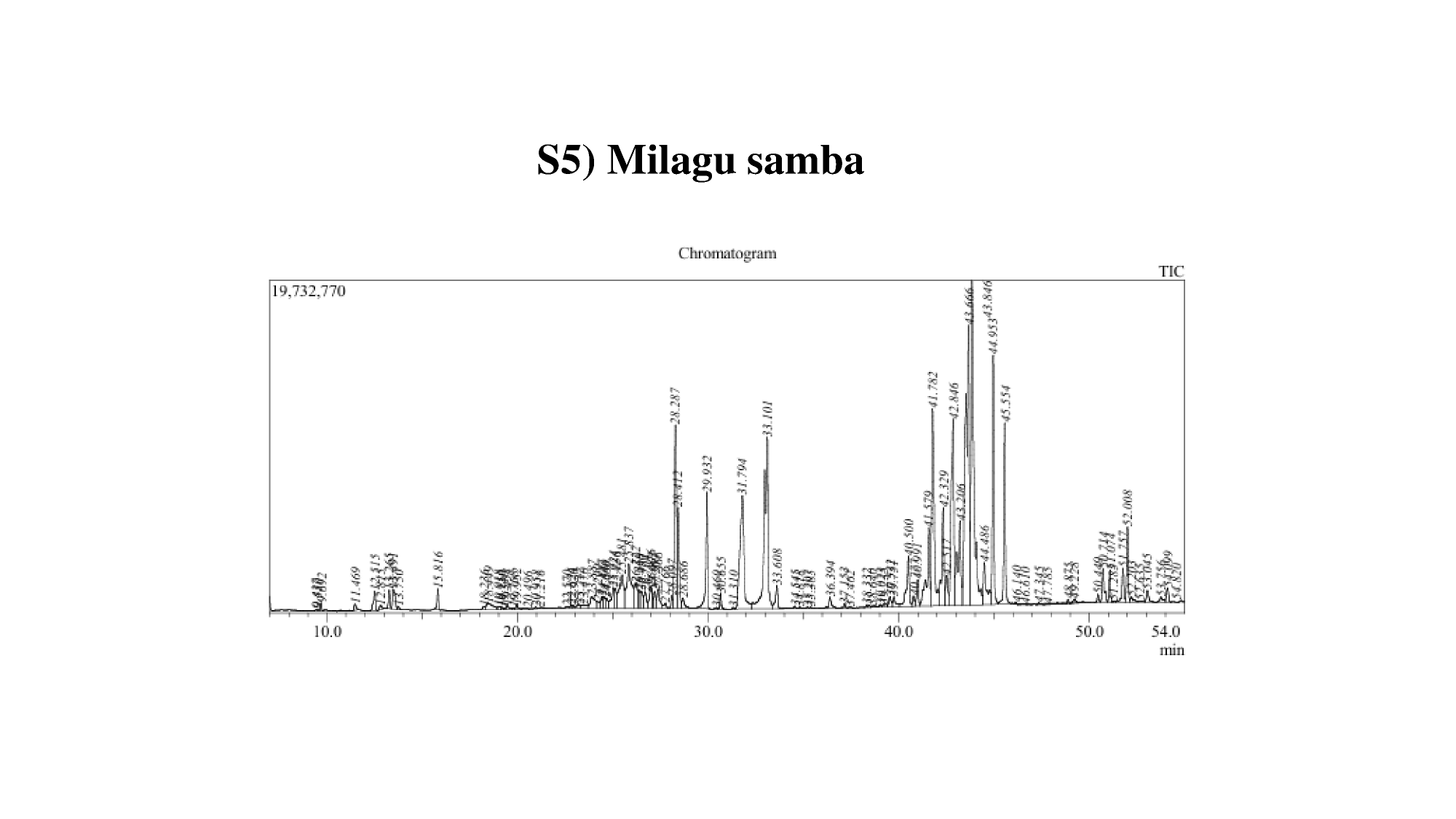

Supplement: Supplementary Figure S5 — GC–MS chromatogram of Milagu Samba. [file Image_5.tiff]

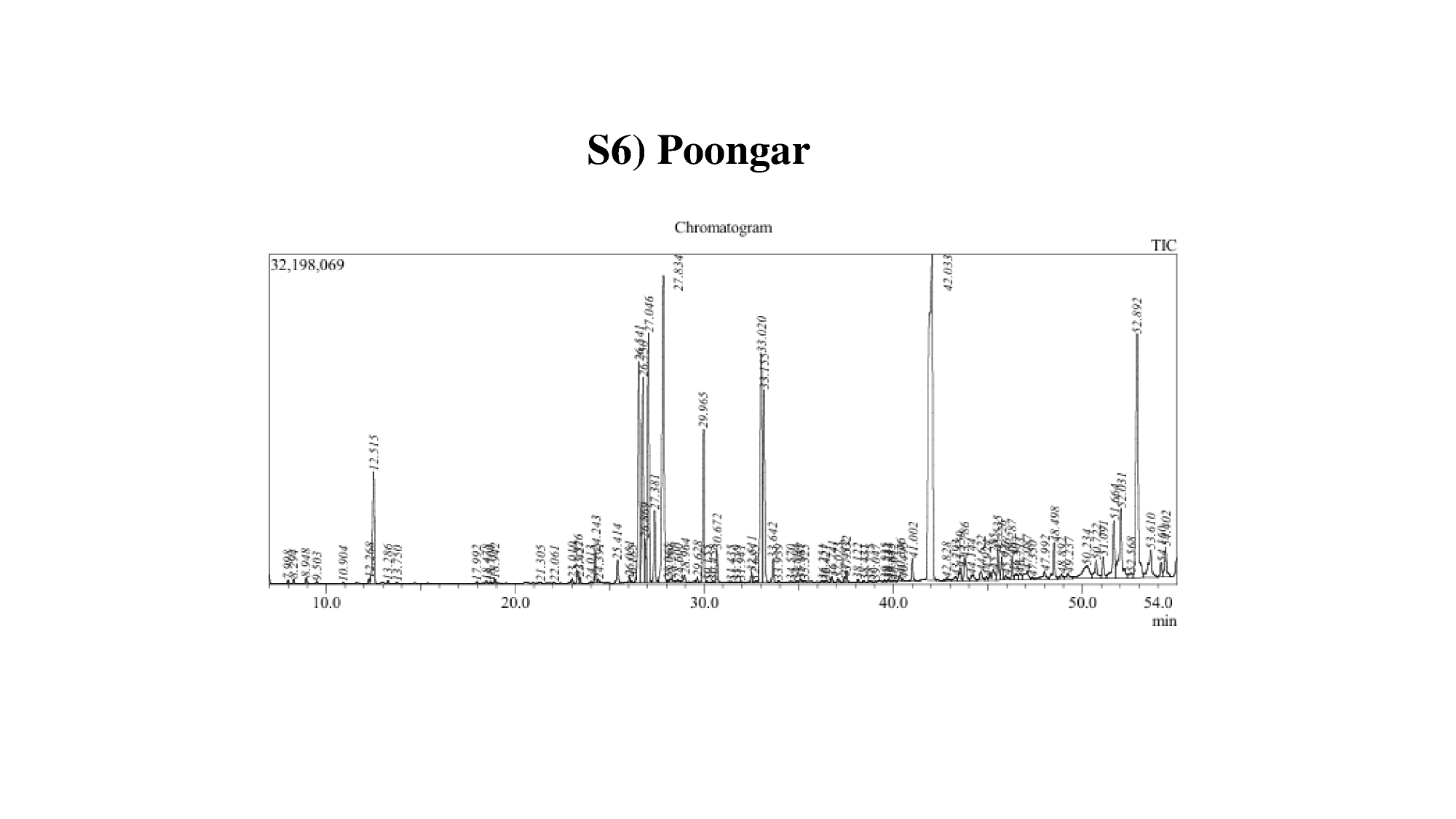

Supplement: Supplementary Figure S6 — GC–MS chromatogram of Poongar. [file Image_6.tiff]
