# Supplementary material for: GC–MS metabolomic profiling and PPARγ-targeted in silico approaches for identifying a potential anti-diabetic compound from traditional rice varieties
Source: Front Nutr. 2026 May 5;13:1800615. doi: 10.3389/fnut.2026.1800615 (PMC13184374; doi:10.3389/fnut.2026.1800615)
Supplement: Supplementary Table S1 — GC–MS analysis of the top 20 compounds identified from traditional rice varieties. [file Table_1.docx]

Supplementary Table S1: GCMS analysis of top 20 compounds from traditional rice variety

| S. No | Name | Pubchem Id | Area | Area % | R.Time | Similarity index with Pioglitazone drug | |
| --- | --- | --- | --- | --- | --- | --- | --- |
|  |  |  |  |  |  | AP Tanimoto | MCS Tanimoto |
| **Annamazhagi** | | | | | | | |
| 1 | Sucrose | 5988 | 755326377 | 38.19 | 42.141 | 0.010 | 0.116 |
| 2 | Linoleic acid | 5280450 | 201478953 | 10.19 | 32.994 | 0.129 | 0.125 |
| 3 | Mannose | 18950 | 104693016 | 5.29 | 27.022 | 0.005 | 0.1562 |
| 4 | Palmitic Acid | 985 | 69990744 | 3.54 | 29.949 | 0.070 | 0.1316 |
| 5 | D-Fructose | 2723872 | 67796225 | 3.43 | 26.513 | 0.008 | 0.156 |
| 6 | 3-Hydroxyvaleric acid | 107802 | 45213934 | 2.29 | 51.676 | 0.025 | 0.178 |
| 7 | Cholest-5-en-3-ol | 5283629 | 41794026 | 2.11 | 52.03 | 0.066 | 0.081 |
| 8 | Glycerol | 753 | 31466726 | 1.59 | 12.52 | 0.003 | 0.148 |
| 9 | Maltose | 439186 | 27679553 | 1.40 | 45.526 | 0.014 | 0.116 |
| 10 | D-(+)-Arabitol | 94154 | 18848384 | 0.95 | 23.324 | 0.002 | 0.129 |
| 11 | Juniperic acid | 10466 | 18630829 | 0.94 | 40.999 | 0.058 | 0.128 |
| 12 | Trehalose | 7427 | 18508496 | 0.94 | 43.574 | 0.009 | 0.116 |
| 13 | 1,3-Dihydroxypropan-2-yl octadec-9-enoate | 543397 | 17897491 | 0.90 | 43.789 | 0.102 | 0.111 |
| 14 | Stearic acid | 5281 | 17067184 | 0.86 | 33.634 | 0.065 | 0.125 |
| 15 | Phosphoric acid | 1004 | 16511501 | 0.83 | 24.258 | 0 | 0.116 |
| 16 | Cholesterol | 5997 | 15040289 | 0.76 | 51.085 | 0.066 | 0.081 |
| 17 | Isomaltose | 439193 | 12984248 | 0.66 | 54.395 | 0.014 | 0.116 |
| 18 | 15 alpha.-Hydroxyculmorin | 23815357 | 10248115 | 0.52 | 54.155 | 0.006 | 0.102 |
| 19 | Inositol phosphate | 107737 | 8236886 | 0.42 | 37.411 | 0.002 | 0.108 |
| 20 | Elaidic acid | 637517 | 7748952 | 0.39 | 50.094 | 0.096 | 0.125 |
| **Karunkuruvai** | | | | | | | |
| 1 | Sucrose | 5988 | 951507027 | 39.40 | 42.198 | 0.010 | 0.116 |
| 2 | 9,12-Octadecadienoic acid (Z,Z) | 5365200 | 141242376 | 5.85 | 32.999 | 0.116 | 0.104 |
| 3 | Mannose | 18950 | 119233539 | 4.94 | 27.035 | 0.005 | 0.156 |
| 4 | Elaidic acid | 637517 | 98423099 | 4.08 | 33.136 | 0.096 | 0.125 |
| 5 | Ribose | 10975657 | 84037847 | 3.48 | 26.726 | 0.005 | 0.166 |
| 6 | D-Fructose | 2723872 | 72483032 | 3.00 | 26.52 | 0.008 | 0.156 |
| 7 | Palmitic Acid | 985 | 66680078 | 2.76 | 29.949 | 0.070 | 0.131 |
| 8 | Cholest-5-en-3-ol, (3.α.) | 5283629 | 64458460 | 2.67 | 52.029 | 0.066 | 0.081 |
| 9 | Acetoacetic acid | 96 | 49557407 | 2.05 | 51.673 | 0.025 | 0.185 |
| 10 | Glycerol | 753 | 42026611 | 1.74 | 12.527 | 0.003 | 0.148 |
| 11 | D-Mannitol | 6251 | 26301366 | 1.09 | 27.762 | 0.005 | 0.121 |
| 12 | Maltitol | 493591 | 24176800 | 1.00 | 53.496 | 0.012 | 0.116 |
| 13 | D-Lactose | 6134 | 21054417 | 0.87 | 45.53 | 0.014 | 0.116 |
| 14 | 1,3-Dihydroxypropan-2-yl octadec-9-enoate, 2 | 543397 | 20738598 | 0.86 | 43.807 | 0.102 | 0.1111 |
| 15 | 7-Hydroxoctanoic acid | 167627 | 19638214 | 0.81 | 45.255 | 0.03 | 0.161 |
| 16 | Isomaltose | 439193 | 19277545 | 0.80 | 54.397 | 0.014 | 0.116 |
| 17 | Stearic acid | 5281 | 15749788 | 0.65 | 33.639 | 0.065 | 0.125 |
| 18 | Phosphoric acid | 1004 | 15316587 | 0.63 | 24.257 | 0 | 0.116 |
| 19 | 1-Monopalmitin | 14900 | 14791686 | 0.61 | 40.998 | 0.080 | 0.116 |
| 20 | 3-α-Mannobiose | 4195243 | 11804864 | 0.49 | 42.862 | 0.009 | 0.116 |
| **Karuppukavuni** | | | | | | | |
| 1 | Sucrose | 5988 | 453135825 | 18.83 | 42.033 | 0.010 | 0.116 |
| 2 | D-Mannitol | 6251 | 227755268 | 9.47 | 27.834 | 0.005 | 0.121 |
| 3 | 9,12-Octadecadienoic acid (Z,Z) | 5365200 | 174153761 | 7.24 | 33.02 | 0.116 | 0.104 |
| 4 | Glucose | 5793 | 149568714 | 6.22 | 27.046 | 0.005 | 0.156 |
| 5 | D-Fructose | 2723872 | 129548594 | 5.38 | 26.541 | 0.008 | 0.156 |
| 6 | Tagatose | 439312 | 122380278 | 5.09 | 26.75 | 0.009 | 0.108 |
| 7 | Elaidic acid | 637517 | 106273485 | 4.42 | 33.155 | 0.096 | 0.125 |
| 8 | Palmitic Acid | 985 | 83375794 | 3.46 | 29.965 | 0.070 | 0.131 |
| 9 | Glycerol | 753 | 70159233 | 2.92 | 12.515 | 0.003 | 0.148 |
| 10 | Cholest-5-en-3-ol, (3.α.) | 5283629 | 69935756 | 2.91 | 52.031 | 0.066 | 0.081 |
| 11 | Acetoacetic acid | 96 | 45538319 | 1.89 | 51.664 | 0.025 | 0.185 |
| 12 | Mannose | 18950 | 36456271 | 1.52 | 27.381 | 0.005 | 0.156 |
| 13 | Oleic acid | 445639 | 35640589 | 1.48 | 50.234 | 0.096 | 0.125 |
| 14 | D-(+)-Turanose | 5460935 | 34339633 | 1.43 | 53.61 | 0.031 | 0.116 |
| 15 | Maltose | 439186 | 24224442 | 1.01 | 54.402 | 0.014 | 0.116 |
| 16 | (Z)-3-[...]-oxyoct-5-enoic acid | 129008953 | 22257430 | 0.92 | 48.498 | 0.072 | 0.119 |
| 17 | Gulonic acid | 152304 | 20706082 | 0.86 | 26.869 | 0.018 | 0.151 |
| 18 | 1,3-Dihydroxypropan-2-yl octadec-9-enoate, 2 | 543397 | 19329244 | 0.80 | 43.786 | 0.102 | 0.111 |
| 19 | D-Lactose | 6134 | 17282787 | 0.72 | 45.535 | 0.014 | 0.116 |
| 20 | Myo-Inositol | 121920 | 16059796 | 0.67 | 30.672 | 0.001 | 0.081 |
| **Mappillai samba** | | | | | | | |
| 1 | Sucrose | 5988 | 695562297 | 31.59 | 42.118 | 0.010 | 0.116 |
| 2 | 9,12-Octadecadienoic acid (Z,Z) | 5365200 | 158501219 | 7.20 | 33.011 | 0.116 | 0.104 |
| 3 | D-Mannitol | 6251 | 111084277 | 5.04 | 27.795 | 0.005 | 0.121 |
| 4 | Elaidic acid | 637517 | 98470475 | 4.47 | 33.144 | 0.096 | 0.125 |
| 5 | Mannose | 18950 | 95877342 | 4.35 | 27.022 | 0.005 | 0.156 |
| 6 | D-(-)-Ribose | 10975657 | 83024794 | 3.77 | 26.726 | 0.005 | 0.166 |
| 7 | Palmitic Acid | 985 | 81923530 | 3.72 | 29.963 | 0.070 | 0.131 |
| 8 | D-Fructose | 2723872 | 66100839 | 3.00 | 26.52 | 0.008 | 0.156 |
| 9 | Glycerol | 753 | 57886517 | 2.63 | 12.514 | 0.003 | 0.148 |
| 10 | 4-Hydroxybutyric acid | 10413 | 45636842 | 2.07 | 51.668 | 0.022 | 0.185 |
| 11 | Cholest-5-en-3-ol, (3.α.) | 5283629 | 27618531 | 1.25 | 52.022 | 0.066 | 0.081 |
| 12 | Mannose | 18950 | 21159001 | 0.96 | 27.364 | 0.005 | 0.156 |
| 13 | D-Lactose | 6134 | 18439038 | 0.84 | 45.53 | 0.014 | 0.116 |
| 14 | 1-Monopalmitin | 14900 | 17599394 | 0.80 | 41.003 | 0.080 | 0.116 |
| 15 | Phosphoric acid | 1004 | 16669007 | 0.76 | 24.252 | 0 | 0.116 |
| 16 | 1,3-Dihydroxypropan-2-yl octadec-9-enoate | 543397 | 16458588 | 0.75 | 43.789 | 0.102 | 0.111 |
| 17 | 7-Hydroxoctanoic acid | 167627 | 14516964 | 0.66 | 45.251 | 0.031 | 0.161 |
| 18 | 12-Bromododecanoic acid | 175468 | 13481900 | 0.61 | 33.64 | 0.054 | 0.142 |
| 19 | Galactose | 6036 | 12788900 | 0.58 | 46.709 | 0.005 | 0.156 |
| 20 | β-Arabinopyranose | 444173 | 3579915 | 0.16 | 36.748 | 0.005 | 0.166 |
| **Milagu samba** | | | | | | | |
| 1 | 2'-Deoxyuridine | 13712 | 231812096 | 12.38 | 43.666 | 0.079 | 0.138 |
| 2 | Maltitol | 493591 | 199061286 | 10.63 | 43.846 | 0.012 | 0.116 |
| 3 | Oleic Acid | 445639 | 139581934 | 7.46 | 33.101 | 0.096 | 0.125 |
| 4 | Trehalose | 7427 | 106410234 | 5.68 | 41.782 | 0.009 | 0.116 |
| 5 | Sucrose | 5988 | 104282597 | 5.57 | 42.846 | 0.010 | 0.116 |
| 6 | 7-Hexadecyn-1-ol | 549047 | 100784963 | 5.38 | 31.794 | 0.023 | 0.105 |
| 7 | 1,5-Anhydro-glucitol | 64960 | 88200721 | 4.71 | 44.953 | 0.008 | 0.161 |
| 8 | β-Arabinopyranose | 444173 | 80780079 | 4.32 | 28.287 | 0.005 | 0.166 |
| 9 | Ribose | 10975657 | 61441016 | 3.28 | 43.206 | 0.005 | 0.166 |
| 10 | Isomaltose | 439193 | 49301730 | 2.63 | 41.579 | 0.014 | 0.116 |
| 11 | Methyl α-D-glucofuranoside | 553466 | 43608990 | 2.33 | 25.837 | 0.010 | 0.102 |
| 12 | Palmitic Acid | 985 | 43233063 | 2.31 | 29.932 | 0.070 | 0.131 |
| 13 | 3-Methyloctanoic acid | 5312327 | 41410641 | 2.21 | 28.412 | 0.041 | 0.161 |
| 14 | Ureidopropionic acid | 111 | 32158544 | 1.72 | 25.481 | 0.040 | 0.214 |
| 15 | 2-Hexenedioic acid | 53440600 | 30211356 | 1.61 | 44.486 | 0.051 | 0.166 |
| 16 | Myristic acid | 11005 | 2919 4347 | 1.56 | 40.5 | 0.060 | 0.138 |
| 17 | Cholest-5-en-3-ol, (3.α.) | 5283629 | 26675130 | 1.43 | 52.008 | 0.066 | 0.081 |
| 18 | 1,1,4,7-Tetramethyldecahydro-1H-cyclopropa[e]azulene-4,7-diol | 178322 | 14765987 | 0.79 | 51.757 | 0.006 | 0.105 |
| 19 | Coniferyl aldehyde | 5280536 | 12883533 | 0.69 | 25.024 | 0.118 | 0.266 |
| 20 | Lignoceric acid | 11197 | 231812096 | 0.58 | 43.666 | 0.050 | 0.108 |
| **Poongar** | | | | | | | |
| 1 | Sucrose | 5988 | 624135130 | 32.67 | 42.094 | 0.010 | 0.116 |
| 2 | 9,12-Octadecadienoic acid (Z,Z) | 5365200 | 111027673 | 5.81 | 32.986 | 0.116 | 0.104 |
| 3 | Mannose | 18950 | 99406158 | 5.20 | 27.022 | 0.005 | 0.156 |
| 4 | D-Fructose | 2723872 | 84810323 | 4.44 | 26.727 | 0.008 | 0.156 |
| 5 | Palmitic Acid | 985 | 64556088 | 3.38 | 29.951 | 0.070 | 0.131 |
| 6 | D-Mannitol | 6251 | 62493845 | 3.27 | 27.779 | 0.005 | 0.121 |
| 7 | Elaidic acid | 637517 | 55693410 | 2.92 | 33.108 | 0.096 | 0.125 |
| 8 | Glycerol | 753 | 40453659 | 2.12 | 12.501 | 0.003 | 0.148 |
| 9 | 4-Hydroxybutyric acid | 10413 | 36588367 | 1.92 | 51.669 | 0.022 | 0.185 |
| 10 | Cholest-5-en-3-ol, (3.α.) | 5283629 | 28440330 | 1.49 | 52.016 | 0.066 | 0.081 |
| 11 | Oleic acid | 445639 | 23144754 | 1.21 | 50.09 | 0.096 | 0.125 |
| 12 | Mannose | 18950 | 21455393 | 1.12 | 27.364 | 0.005 | 0.156 |
| 13 | Isomaltose | 439193 | 16758762 | 0.88 | 53.511 | 0.014 | 0.116 |
| 14 | Maltose | 439186 | 16526089 | 0.87 | 45.53 | 0.0146 | 0.116 |
| 15 | 1-Monopalmitin | 14900 | 14046966 | 0.74 | 41 | 0.080 | 0.116 |
| 16 | 7-Hydroxoctanoic acid | 167627 | 13907779 | 0.73 | 45.251 | 0.031 | 0.161 |
| 17 | Phosphoric acid | 1004 | 13019539 | 0.68 | 24.251 | 0 | 0.116 |
| 18 | 12-Bromododecanoic acid | 175468 | 11691726 | 0.61 | 33.632 | 0.054 | 0.142 |
| 19 | Lactose | 440995 | 11654648 | 0.61 | 42.81 | 0.014 | 0.116 |
| 20 | Margaric acid | 10465 | 10696699 | 0.56 | 47.992 | 0.072 | 0.128 |
