# Supplementary material for: GC–MS metabolomic profiling and PPARγ-targeted in silico approaches for identifying a potential anti-diabetic compound from traditional rice varieties
Source: Front Nutr. 2026 May 5;13:1800615. doi: 10.3389/fnut.2026.1800615 (PMC13184374; doi:10.3389/fnut.2026.1800615)
Supplement: Supplementary Table S2 — Molecular docking results of 53 compounds, along with the reference drug pioglitazone. [file Table_2.docx]

Supplementary Table S2:Molecular docking and binding free energy results of 53 compounds along with the reference drug pioglitazone

| S.No | Pubchem_Id | Compound name | PyRx binding affinity | MMGBSA |
| --- | --- | --- | --- | --- |
|  | 445639 | Oleic Acid | -7.6 | -108.2193314 |
|  | 5997 | Cholesterol | -7.4 | -65.9472725 |
|  | 5283629 | Cholest-5-en-3-ol | -7.2 | -59.68684852 |
|  | 178322 | 1,1,4,7-Tetramethyldecahydro-1H-cyclopropa[e]azulene-4,7-diol | -7.1 | -53.28250054 |
|  | 439186 | Maltose | -6.9 | -61.67036086 |
|  | 13712 | 2-Deoxyuridine | -6.8 | -58.6043861 |
|  | 439193 | Isomaltose | -6.6 | -54.37593706 |
|  | 637517 | Elaidic acid | -6.4 | -87.63843139 |
|  | 11197 | Lignoceric acid | -6.2 | -104.1686524 |
|  | 5280536 | Coniferyl aldehyde | -6.2 | -50.99935614 |
|  | 4195243 | 3-α-Mannobiose | -6.1 | -72.45986209 |
|  | 4829(drug) | Pioglitazone | -5.9 | -96.81136089 |
|  | 6134 | D-Lactose | -5.9 | -54.58703231 |
|  | 543397 | 1,3-Dihydroxypropan-2-yl octadec-9-enoate | -5.9 | -104.4190855 |
|  | 14900 | 1-Monopalmitin | -5.8 | -106.4935614 |
|  | 440995 | Lactose | -5.8 | -54.72818417 |
|  | 5365200 | 9,12-Octadecadienoic acid (Z,Z)-, octyl ester | -5.8 | -92.50873961 |
|  | 549047 | 7-Hexadecyn-1-ol | -5.8 | -74.6288674 |
|  | 5988 | Sucrose | -5.7 | -54.19580364 |
|  | 10465 | Margaric acid | -5.7 | -93.08601597 |
|  | 107737 | Inositol Phosphates | -5.7 | -47.36252528 |
|  | 7427 | Trehalose | -5.6 | -52.58703162 |
|  | 5281 | Stearic acid | -5.6 | -92.64017902 |
|  | 10466 | Juniperic acid | -5.6 | -87.8724491 |
|  | 11005 | Myristic acid | -5.6 | -78.82235859 |
|  | 6036 | Galactose | -5.6 | -32.96806294 |
|  | 175468 | 12-Bromododecanoic acid | -5.5 | -78.02085093 |
|  | 985 | Palmitic Acid | -5.4 | -80.33839434 |
|  | 5460935 | Turanose | -5.3 | -47.04027104 |
|  | 493591 | Maltitol | -5.3 | -72.2875475 |
|  | 5280450 | Linoleic acid | -5.3 | -92.93598441 |
|  | 5793 | D-Glucose | -5.3 | -44.71665504 |
|  | 18950 | Mannose | -5.3 | -37.37106341 |
|  | 152304 | D-Gulonic acid | -5.2 | -41.50246983 |
|  | 121920 | Myo-Inositol | -5.2 | 3.552082275 |
|  | 2723872 | D-Fructose | -5 | -42.66286647 |
|  | 111 | Ureidopropionic acid | -4.9 | -30.14212709 |
|  | 64960 | 1,5-Anhydroglucitol | -4.9 | -32.55764765 |
|  | 439312 | D-Tagatose | -4.8 | -42.92641211 |
|  | 5312327 | 3-Methyloctanoic acid | -4.8 | -45.9785646 |
|  | 10975657 | D-Ribose | -4.8 | -43.97201348 |
|  | 53440600 | 2-Hexenedioic acid | -4.8 | -27.44176956 |
|  | 107802 | 3-Hydroxyvaleric acid | -4.8 | -34.23894542 |
|  | 444173 | beta-D-arabinopyranose | -4.8 | -38.3321399 |
|  | 6251 | Mannitol | -4.8 | -37.47157487 |
|  | 553466 | Methyl α-D-glucofuranoside | -4.6 | -58.0435854 |
|  | 94154 | D-(+)-Arabitol | -4.5 | -38.99142667 |
|  | 96 | Acetoacetic acid | -4.1 | -29.12876801 |
|  | 10413 | 4-Hydroxybutyric acid | -3.9 | -31.69968474 |
|  | 129008953 | (Z)-3-[(2R,3R,4S,5S,6R)-3,4,5-Trihydroxy-6-(hydroxymethyl)oxan-2-yl]oxyoct-5-enoic acid | -3.8 | -82.30871062 |
|  | 753 | Glycerol | -3.5 | -38.3066247 |
|  | 167627 | 7-Hydroxoctanoic acid-2 | -3.4 | -51.33571029 |
|  | 1004 | Phosphoric acid | -3.4 | -12.70087597 |
|  | 23815357 | 15alpha-Hydroxyculmorin | -3.2 | -9.178685404 |
